# FSTL3 promotes tumor immune evasion and attenuates response to anti-PD1 therapy by stabilizing c-Myc in colorectal cancer

**DOI:** 10.1038/s41419-024-06469-0

**Published:** 2024-02-01

**Authors:** Haiyang Li, Na Zheng, Anning Guo, Weiwei Tang, Muxin Li, Yuanyuan Cao, Xinhua Ma, Hongyong Cao, Yong Ma, Hanjin Wang, Shuli Zhao

**Affiliations:** 1 Department of general surgery, Nanjing First Hospital, Nanjing Medical University, Nanjing, Jiangsu China; 2 General Clinical Research Center, Nanjing First Hospital, Nanjing Medical University, Nanjing, Jiangsu China; 3 General Clinical Research Center, Nanjing First Hospital, China Pharmaceutical University, Nanjing, Jiangsu China; 4 Hepatobiliary/Liver Transplantation Center, the First Affiliated Hospital of Nanjing Medical University, Key Laboratory of Living Donor Transplantation, Chinese Academy of Medical Sciences, Nanjing, Jiangsu China

**Keywords:** Cancer microenvironment, Cancer immunotherapy, Cancer therapeutic resistance

## Abstract

Programmed cell death 1 ligand 1 (PDL1)/programmed cell death 1 (PD1) blockade immunotherapy provides a prospective strategy for the treatment of colorectal cancer (CRC), but various constraints on the effectiveness of the treatment are still remaining. As reported in previous studies, follistatin-like 3 (FSTL3) could mediate inflammatory response in macrophages by induction lipid accumulation. Herein, we revealed that FSTL3 were overexpressed in malignant cells in the CRC microenvironment, notably, the expression level of FSTL3 was related to tumor immune evasion and the clinical efficacy of anti-PD1 therapy. Further studies determined that hypoxic tumor microenvironment induced the FSTL3 expression via HIF1α in CRC cells, FSTL3 could bind to the transcription factor c-Myc (354–406 amino acids) to suppress the latter’s ubiquitination and increase its stability, thereby to up-regulated the expression of PDL1 and indoleamine 2,3-dioxygenase 1 (IDO1). The results in the immunocompetent tumor models verified that FSLT3 knockout in tumor cells increased the proportion of CD8^+^ T cells in the tumor microenvironment, reduced the proportion of regulatory T cells (CD25^+^ Foxp3^+^) and exhausted T cells (PD1^+^ CD8^+^), and synergistically improved the anti-PD1 therapy efficacy. To sum up, FSTL3 enhanced c-Myc-mediated transcriptional regulation to promote immune evasion and attenuates response to anti-PD1 therapy in CRC, suggesting the potential of FSTL3 as a biomarker of immunotherapeutic efficacy as well as a novel immunotherapeutic target in CRC.

## Introduction

On account of dietary patterns, obesity and lifestyle factors, colorectal cancer (CRC) has become the leading gastrointestinal malignancy in terms of incidence and mortality worldwide [1]. The process that leads to the progression of CRC involves the accumulation of mutations in several oncogenes at various stages, and the immunological microenvironment in which the tumor is implanted also plays a pivotal regulatory role [2]. Due to the crucial role of immune system in disease defense and surveillance, the immunotherapy that targets the immune system has become the forefront of cancer treatment in recent years, opening up new avenues for the treatment of CRC [3].

Concerning the immunotherapy of CRC, there is a considerable advantage demonstrated by the immune checkpoint blockade (ICB) that targets immune checkpoints such as programmed cell death 1 (PD1), programmed cell death 1 ligand 1 (PDL1), and cytotoxic T lymphocyte-associated antigen 4 (CTLA-4) [4]. However, microsatellite instability-high (MSI-H) patients account for the majority of CRC patients responding to ICB, while MSI-H patients account for merely around 15% of all CRC cases. Besides, the majority of patients with microsatellite instability-low (MSI-L) and 50% of MSI-H patients present with resistance to ICB [5–7]. It is suspected that insufficient tumor antigen presentation, T cell exhaustion, and immunosuppressive signaling in the tumor microenvironment (TME) all contribute to the ICB resistance in CRC [2]. Therefore, it is imperative to fully understand the defensive and escape mechanisms of cancer cells against immune system, which is a precondition to exploring the potential biomarkers needed to identify populations potentially responsive to immunotherapy, and to discovering new immunotherapy targets.

Follistatin-like 3 (FSTL3) refers to a secreted glycoprotein released by adipose tissue, reproductive system, pancreas, liver, skeletal muscle and placenta [8]. In addition to regulating testicular senescence and growth, FSTL3 also gets involved in the regulation of glucose and lipid homeostasis [9]. FSTL3 has been discovered to promote lipid buildup and the release of inflammatory cytokines, as well as trigger the expression of CD36 and lipoxygenase 1 in foam cells [10]. FSTL3 has also been demonstrated as conducive to the development of lung, kidney and gastric cancer, and applicable as a novel marker of poor survival [11–13]. According to some studies, FSTL3 is highly expressed in CRC tissues and is associated with the poor prognosis of CRC patients [14–16]. However, there remains a lack of clarity on the function and mechanisms of FSTL3 in immune evasion and immunotherapy response in CRC.

In the present study, it is revealed that FSTL3 is hyper-expressed in cancer cells of CRC. The ablation of FSTL3 in CRC has a promoting effect on the creation of an anti-tumor microenvironment, which is mainly characterized by the improved anti-tumor efficacy of CD8^+^ T cells and repressed infiltration of regulatory T cells (Tregs). Besides, the hyperexpression of FSTL3 could prompt the resistance of mouse tumors to anti-PD1 therapy. This study reveals the exact role of FSTL3 in immune evasion and ICB therapy resistance in CRC, which is essential for finding a new biomarker and therapeutic pathway for those CRC patients resistant to ICB therapy.

## Materials and methods

### Patient tissue samples

In the present study, normal colorectal tissues (*n* = 35) and CRC tissues (*n* = 95) were derived from those CRC patients who underwent surgery and were diagnosed by histopathology. The above samples were collected between May 2019 and May 2022 from Nanjing First Hospital, Nanjing Medical University. The patients received no chemotherapy or radiotherapy prior to surgical resection. All samples were accessed with the informed consent of patients, as approved by the Nanjing First Hospital, Nanjing Medical University Ethics Committee.

### Datasets gathering and processing

The gene expression data, somatic mutation data and clinical information of The Cancer Genome Atlas (TCGA) CRC cohort were sourced from TCGA database (https://portal.gdc.cancer.gov). Neoantigen data and microsatellite instability data of TCGA CRC cohort were collected from the supplementary files of Thorsson’s study and Roudko’s study respectively [17, 18]. The analysis of the cancer-immunity cycle was performed on the TIP platform (http://biocc.hrbmu.edu.cn/TIP/) [19]. The analysis of immune cell infiltration was performed on the TIMER2.0 platform (http://timer.cistrome.org/) [20]. The CRC datasets (GSE44076, GSE21510, GSE41657, and GSE39582), the dataset of hypoxia-treated human colorectal cancer cells RKO (GSE145108), and the datasets of ICB therapy cohorts (GSE91061 and GSE78220) were downloaded from the NCBI Gene Expression Omnibus (GEO) database (https://www.ncbi.nlm.nih.gov/geo/). Additionally, In addition, the other two ICB therapy cohorts (PMID29301960 and PMID32472114) were supplemented from Miao’s study and Braun’s study respectively [21, 22].

### Single cell RNA sequencing analysis

Single cell sequencing analysis was performed as described previously [23].

### Immunohistochemical (IHC) staining

The tissue was formalin-fixed, paraffin-embedded, and cut into 3–5 μm slides. Then, the slides were deparaffinized with dimethylbenzene and graded alcohol, followed by an epitope recovery process conducted in the citrate buffer solution as thermally induced. After being blocked with 5% BSA and hatched with primary antibody, these slides were hatched with HRP-labeled goat anti-rabbit IgG antibody. Afterwards, immunostaining was assessed against 3,3′-diaminobenzidine and hematoxylin. The antibodies used for IHC are listed in Supplementary Table S2.

### Immunofluorescence (IF) staining

To begin with, these slides were fixed with 4% paraformaldehyde and then permeabilized with 0.1% Triton X-100. Secondly, they were blocked with 10% goat serum and incubated overnight at 4 °C with primary antibody. Thirdly, the slides were washed in PBS, stained with fluorophore-coupled secondary antibodies for 1 h at room temperature, and then stained with DAPI. Finally, the slides were observed under fluorescence microscopy. The antibodies used for IF are listed in Supplementary Table S2.

### Western blotting

The protein was extracted from lysed cells, quantified, isolated by sodium dodecyl sulfate-polyacrylamide gel electrophoresis (SDS-PAGE), and then transferred to PVDF membranes (Millipore, USA). The protein-containing membranes were blocked with 5% skim milk, incubated overnight at 4 °C with primary antibody, and then further incubated with secondary antibody. Finally, the protein signal was examined with the assistance of a chemiluminescence reagent kit (New Cell and Molecular Biotech, Suzhou, China). The antibodies used for western blotting are listed in Supplemental Table S2.

### Cell culture and transfection

Human CRC cell lines (HCT116 and HCT8) and mouse CRC cell line (MC38) were all purchased from Type Culture Collection of Chinese Academy of Sciences (Shanghai, China). HCT116 and HCT8 cells were cultured in the DMEM media supplemented with 10% FBS and 1% penicillin–streptomycin. MC38 cells were cultured in the RPMI1640 medium containing 10% FBS and 1% penicillin–streptomycin.

The transfection of plasmids or siRNA into cells was performed by using Lipofectamine 3000 as instructed by the manufacturer. According to the MOI values, the cells were infected with lentivirus and then treated with puromycin (2 μg/mL) to identify those consistently transfected cells. Short tandem repeat DNA profiling was used to identify all cell lines and check for mycoplasma contamination.

### Plasmids, siRNA and lentivirus

To construct the expression plasmids, human full-length FSTL3, FSTL3-(36–107aa) deletion, FSTL3-(99–119aa) deletion, FSTL3-(113–169aa) deletion and FSTL3-(189–245aa) deletion cDNA were cloned respectively into the pCMV-Tag 2B vector (3×Flag-tag). Full-length human c-Myc, c-Myc-(204–295aa) deletion, c-Myc-(354–406aa) deletion and c-Myc-(413–434aa) deletion cDNA were cloned respectively into the pCMV-Tag 2B vector (HA-tag). Human full-length HIF1α and human full-length c-Myc were cloned respectively into the pcDNA vector. The above plasmids were provided by GENECHEM (Shanghai, China). The siRNA targeting c-Myc was sourced from KeyGene Biotech (Nanjing, China). The knockdown lentiviruses expressing shRNAs targeting FSTL3 (shFSTL3) and negative control shRNA (shNC) were constructed through GV248 lentiviral vector. The overexpression lentiviruses expressing FSTL3^Flag^ were constructed through GV502 lentiviral vector. All lentiviruses were provided by GENECHEM. The target sequences for siRNAs and shRNAs are detailed in Supplementary Table S3.

### RNA extraction and quantitative real-time PCR (qRT-PCR)

Total RNA was extracted from the tissue or cells using TRIzol reagent (Invitrogen, Frederick, MD, USA). In line with the instructions of the manufacturer, reverse transcription was performed with HiScript II Q RT SuperMix for qPCR (+gDNA wiper) (Vazyme, Nanjing, China), and qRT-PCR was conducted by using the ChamQ Universal SYBR qPCR Master Mix kit (Vazyme). The primers used for quantitative qRT-PCR are listed in Supplemental Table S4.

### Chromatin immunoprecipitation (ChIP) assays

ChIP assay was conducted by using the SimpleChIP Enzymatic Chromatin IP Kit (Cell Signaling Technology, USA) in accordance with the protocol formulated by the manufacturer. In brief, the cells were cross-linked with formaldehyde, lysed with sodium dodecyl sulfate buffer, and then sonicated. Following sonication, the fragmented chromatin was added into ChIP dilution buffer and incubated overnight with primary antibody. IgG antibody (Cell Signaling Technology) was taken as a negative control and Histone H3 antibody (Cell Signaling Technology) was taken as a positive control. Immunoprecipitation product was gathered following the incubation with ChIP-Grade Protein G Magnetic Beads (Cell Signaling Technology). After the binding chromatin was eluted and digested with Proteinase K (Cell Signaling Technology), the purified DNA was applied to conduct qRT-PCR assay. Supplemental Table S2 lists the antibodies used for ChIP and Supplementary Table S4 shows the sequences of forward and reverse primers used for ChIP.

### Colony formation assay

The cells were inoculated at 1000 per well into 6 cm dishes and incubated for 2 weeks. Afterwards, the colonies were fixated with 4% paraformaldehyde, dyed with crystal violet and observed for counting.

### EdU incorporation assay

Cell proliferation activity was measured by using the EdU cell proliferation assay kit (KeyGene Biotech) in line with the instructions of the manufacturer. The images were captured by fluorescence microscopy. The ratio of EdU-positive stained cells to Hoechst-stained cells per well was defined as the cell proliferation rate.

### T cells isolation and co-culture

CD8^+^ T cells and CD4^+^ T cells were separated from human peripheral blood using the magnetic cell separation system, human CD8^+^ T cell separation kit (Miltenyi Biotec, Friedrich, Germany) and human CD4^+^ T cell separation kit (Miltenyi Biotec). The isolated T cells were co-cultured with human CRC cells at the indicated ratio in 12-well plates.

### Flow cytometry

For the analysis of immune cell populations in mouse tumors, the tumors were minced mechanically into small pieces using scissors. Then, these small pieces were digested in RPMI 1640 medium containing 10% FBS, 1% penicillin–streptomycin, 0.05 mg/mL collagenase type I (Sigma-Aldrich, USA), 0.05 mg/mL collagenase type IV (Sigma-Aldrich), 0.025 mg/mL hyaluronidase (Sigma-Aldrich) and 0.01 mg/mL DNase I (Roche) for 30 min. Post-digestion samples were sifted through 70 µM sieves and the red blood cells (RBCs) were lysed with Red Blood Cell Lysis Buffer (Beyotime Biotechnology, Shanghai, China). Cell labeling was performed with the fluorescently conjugated antibodies directed against mouse CD45, CD3, CD8, CD4, PD1, Foxp3, CD25, and interferon gamma (IFNγ). The fixation/permeabilization solution kit (eBioscience, USA) was applied to carry out intracellular staining. Flow cytometric analysis was conducted on DxFLEX flow cytometer (Beckman) and the relevant data were subsequently analyzed with FlowJo V10.

To analyze the PDL1 expression of HCT116 and HCT8 cells, these cells were washed, separated into single cells, and stained with fluorescently conjugated anti-PDL1 antibody. Finally, these cells were examined by means of flow cytometry.

To analyze the proliferation and apoptosis of CD8^+^ T cells, isolated CD8^+^ T cells were cultured in RPMI 1640 with 10% FBS and 1 ng/ml IL-2 and activated with anti-CD3/CD28 antibody, and a certain proportion of CD8^+^ T cells were labeled with CFSE (BD Pharmingen, USA) prior to culturing. After culturing, the apoptosis of unlabeled-CFSE CD8^+^ T cells treated with the apoptosis detection kit (KeyGene Biotech) and the proliferation of CFSE-labeled CD8^+^ T cells were assessed through flow cytometric analysis.

To determine the proportion of Tregs, the isolated CD4^+^ T cells were cultured in the RPMI 1640 medium containing 10% FBS, 1 ng/ml IL-2 and 5 ng/ml TGF-β and then activated with anti-CD3/CD28 antibody. The cells were labeled with CD25 and Foxp3 and detected by flow cytometry. The antibodies used for flow cytometry are listed in Supplemental Table S5.

### RNA-sequencing analysis

The total RNA extracted from HCT116 cells was transfected with shFSTL3 and the control cells were lysed with TRIzol reagent. The cDNA library was constructed after the RNA samples were qualified. Immediately after the library was examined and passed, Illumina high-throughput sequencing was performed on the machine. The raw data of sequencing were evaluated for their quality and then compared with the reference genome for analysis. Finally, the gene expression data were collected. The above RNA-sequencing was performed by Aptbiotech Corp (Shanghai, China). The analysis of differentially expressed genes (DEGs) and Gene Ontology (GO) enrichment was carried out using R v3.6.1 (https://www.r-project.org/).

### Co-immunoprecipitation (Co-IP) and liquid chromatography-tandem mass spectrometry (LC–MS/MS)

The supernatant was obtained by lysing the cells with the NP-40 lysis buffer (KeyGene Biotech) containing phosphatase and protease inhibitors. Then, the supernatant was mixed with anti-Flag, anti-c-Myc, or anti-HA antibody at 4 °C overnight. The protein A/G magnetic beads (MedChemExpress, USA) were subsequently co-incubated with the protein antibody complex for 4 h. After co-incubation, the complexes were washed four times with lysis buffer, followed by western blotting to examine the efficiency of immunoprecipitation and the proteins interacting with the corresponding antibodies. The antibodies used for immunoprecipitation are listed in Supplemental Table S2.

To conduct mass spectrometry analysis, the eluted samples were separated on a 10% gel and stained with the rapid silver staining kit (Beyotime Biotechnology) for silver staining. Subsequently, the silver-stained gel strips were analyzed with LC-MS/MS, and the data were compared against the UniProtKB/Swiss-Prot human database using MaxQuant software.

### Ubiquitination assay

The cells were lysed after 6 h of treatment with 10 μM MG132. Cell lysate was immunoprecipitated with anti-c-Myc antibody at 4 °C overnight and the level of ubiquitination was further examined by means of western blotting.

### Animal studies

All animal experiments were approved by the animal care committee of Nanjing First Hospital, Nanjing Medial University (ethical numbers: DWSY-22167294). All the animals involved were housed in a specific pathogen free environment at appropriate temperatures. In this study, mouse subcutaneous tumor models were constructed by using C57BL/6J and BABL/c-nude female mice (6 weeks old). Lentivirus-mediated shFSTL3 and FSTL3^Flag^ cDNA were delivered to MC38 cells, so as to establish steady FSTL3 knockdown (FSTL3-KD) and FSTL3 overexpressing (FSTL3-OE) MC38 cells.

To investigate the role of FSTL3 in MC38 cells in CRC microenvironment, C57BL/6J or BABL/c-nude female mice were divided into two groups each (*n* = 5 each group). One was FSTL3-KD group, subcutaneously injected with 2 × 10^6^ FSTL3-KD MC38 cells. The other was NC-KD group, subcutaneously injected with 2 × 10^6^ NC-KD MC38 cells.

To investigate the role of hyper-expressed FSTL3 in CRC immunotherapy, C57BL/6J female mice were divided into four groups (*n* = 5 each). The first one was FSTL3-OE + PBS group, subcutaneously injected with 2 × 10^6^ FSTL3-OE MC38 cells followed by PBS. The second one was FSTL3-OE+anti-PD1 group, subcutaneously injected with 2 × 10^6^ FSTL3-OE MC38 cells, followed by anti-PD1 antibody (anti-mouse PD1, Bio X cell, USA). The third one was NC-OE + PBS group, subcutaneous injection of 2 × 10^6^ NC-OE MC38 cells, followed by PBS. The last one was NC-OE+anti-PD1 group, subcutaneous injection of 2 × 10^6^ NC-OE MC38 cells, followed by anti-PD1 antibody.

To investigate the role of IDO1 inhibitor (1-methyl-L-tryptophan, 1-MT, Sigma-Aldrich) combined with anti-PD1 treatment in FSTL3 overexpressing tumors, 2 × 10^6^ FSTL3-OE MC38 cells were injected subcutaneously into C57BL/6J mice. After the tumor size reached 5 × 5 mm, the mice were randomly divided into 4 groups (*n* = 5 each group). The first one was PBS group, given PBS. The second one was anti-PD1 group, given anti-PD1 antibody. The third one was 1-MT group, given 1-MT. The last one was anti-PD1 + 1-MT group, given a combination of 1-MT and anti-PD1 antibody.

To investigate the role of HIF1α inhibitor (BAY87-2243, MedChemExpress) in FSTL3 overexpressing tumors, 2 × 10^6^ FSTL3-OE MC38 cells were injected subcutaneously into C57BL/6J mice. After the tumor size reached 5 × 5 mm, the mice were randomly divided into 2 groups (*n* = 5 each group). The first one was PBS group, given PBS. The second one was BAY87-2243 group, given BAY87-2243.

The mice were administered the following drugs: IDO1 inhibitor (1-MT, 5 mg/mL in drinking water with sweetener, 3-4 mL/mouse/day), anti-PD1 antibody (100 μg/mouse intraperitoneally on day 7, 10 and 13), combination treatment (same dose and date as single treatment) and HIF1α inhibitor (BAY87-2243, 4 mg/kg/every other day administered orally). The control mice were given equal amounts of PBS to simulate the treatment. The tumor volumes of mice were recorded every 3 days. The formula used for the calculation of tumor volume was 0.52 × length × width^2^.

### Enzyme-linked immunosorbent assay (ELISA)

As instructed by the manufacturer, the concentrations of kynurenine and tryptophan in MC38 cell supernatants were determined by using the Mouse kynurenine ELISA Kit (Yanjin Biology Company, Shanghai, China) and the Mouse tryptophan ELISA Kit (Yanjin Biology Company).

### Statistical analysis

Statistical analyses were conducted by using GraphPad Prism 8.0 software. The data were obtained and indicated as the mean ± SD. For comparison between the two groups, two-sided Student’s *t*-test or Mann–Whitney *U*-test was conducted as appropriate. For the comparison between three or more groups, the one-way analysis of variance (ANOVA) was carried out. The correlation between FSTL3 expression and clinicopathological variables in CRC patients was statistically tested through the Pearson *χ*^2^ test or Fisher’s exact test as appropriate. Spearman’s rank correlation was applied to examine the correlation between the two variables. A *P* value < 0.05 was treated as statistically significant. Where indicated, individual *P* value was displayed, alternatively the following symbols were used to describe statistical significance: *P < 0.05, **P < 0.01, ***P < 0.001, ns, non-significant.

## Results

### FSTL3 is hyper-expressed in cancer cells and closely correlates with immune evasion of CRC

**Fig. 1:**
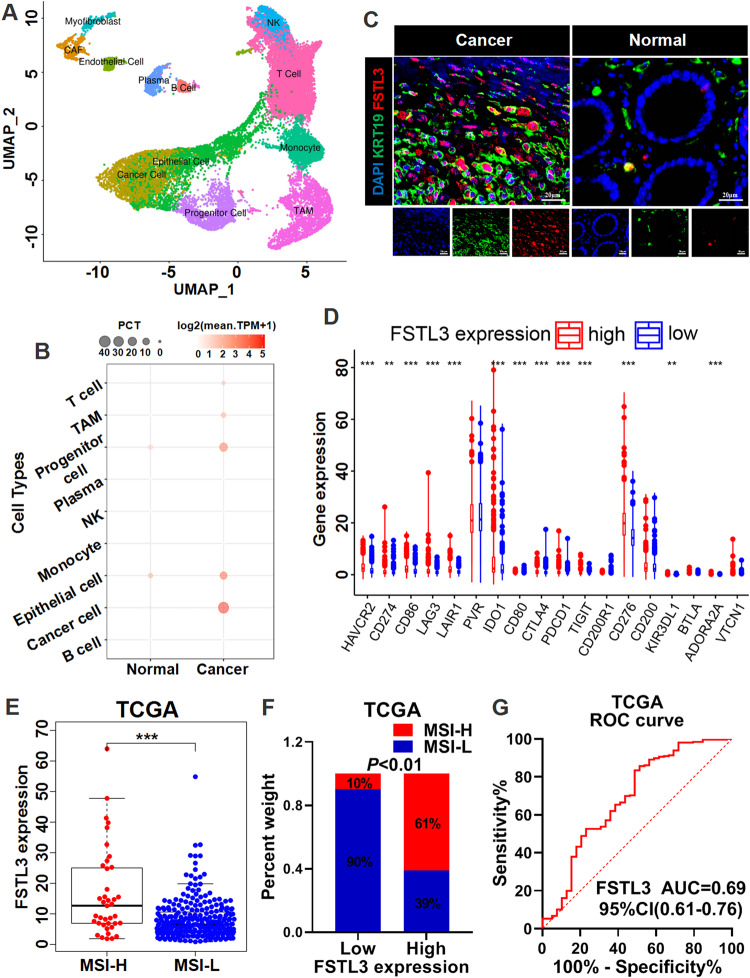
FSTL3 is hyper-expressed in cancer cells and closely correlates with microsatellite instability of CRC. **A** The single-cell sequencing analysis identified 12 cell clusters in CRC tissues including mainly tumor-associated macrophage (TAM), cancer cell, monocyte, cancer-associated fibroblast, epithelial cell, T cell, NK cell, and B cell and other cell clusters. **B** The expression of FSTL3 in different cell clusters in CRC tissues and normal tissues. **C** Immunofluorescence analysis of FSTL3 expression in KRT19-positive cells (cancer cells) of CRC tissues. **D** Expression of immune checkpoints in high versus low FSTL3 expression subgroups. **E** The expression level of FSTL3 in microsatellite instability-high (MSI-H) tumors and microsatellite instability-low (MSI-L) tumors. **F** The proportion of MSI-H tumors in high versus low FSTL3 expression subgroups. **G** ROC curve showing the predictive efficiency of FSTL3 expression for microsatellite instability. Data are shown as mean ± SD. **P < 0.01, ***P < 0.001.

In order to explore the relevance of FSTL3 in CRC microenvironment, single-cell transcriptome sequencing was performed for primary CRC tissues and adjacent normal intestinal tissues. With the cell cluster classification defined using cell type markers in line with the Uniform Manifold Approximation and Projection (UMAP) plots, a total of 12 cell clusters were categorized, including tumor-associated macrophage (TAM), cancer cell, monocyte, cancer-associated fibroblast (CAF), epithelial cell, T cell, NK cell, and B cell and other cell clusters (Fig. 1A). Among these cell type markers of cell clusters, cancer cell cluster specifically expressed keratin 19 (KRT19) and CEA cell adhesion molecule 1 (CEACAM1), and TAM cluster expressed CD68, CD163, and complement C1q C chain (C1QC) (Supplementary Fig. S1A). According to Fig. 1B, the cancer cell cluster of CRC tissues exhibited the highest expression level of FSTL3 among the above cell clusters, and the expression of FSTL3 there was significantly up-regulated compared to the normal tissues. Furthermore, the IF analysis of CRC tissues revealed that a significant FSTL3 expression was shown by KRT19-positive cancer cells (Fig. 1C and Supplementary Fig. S1B).

In comparison with the matched normal samples, the cancer tissues in the TCGA CRC cohort exhibited higher levels of FSTL3 expression (Supplementary Fig. S2A). An identical pattern was observed in the data of GEO database (Supplementary Fig. S2B). Afterwards, western blotting and IHC were performed to analyze the expression of FSTL3 protein in clinical CRC tissues. According to the analytical results, the level of FSTL3 protein expression was higher in the cancer tissues than in the normal tissues, and the hyperexpression of FSTL3 occurred predominantly in the nucleus (Supplementary Fig. S2C, D). Also, a higher level of FSTL3 expression was manifested by the more advanced T-stage CRC tissues (Supplementary Fig. S2E). Meanwhile, according to the analysis of correlation between the level of FSTL3 expression and clinicopathological characteristics in clinical specimens, those patients with high FSTL3 expression were in more advanced T, N, M and TNM stages, with a larger tumor diameter (Supplementary Table S1). As shown in Supplementary Fig. S2F, the CRC patients with high FSTL3 expression had a substantially shorter overall survival than those with low FSTL3 expression.

Upon integrating data on gene expression, tumor mutations and neoantigens from the TCGA CRC cohort, the analysis revealed that the immune checkpoints were significantly hyper-expressed in the group with high FSTL3 expression, including PDL1 (CD274), indoleamine 2,3-dioxygenase 1 (IDO1), CD80, PD1 (PDCD1), T cell immunoreceptor with Ig and ITIM domains (TIGIT) and CD276 (Fig. 1D). Meanwhile, tumor mutation burden (TMB) and neoantigen load were significantly elevated in the group with high FSTL3 expression (Supplementary Fig. S3A-B). MSI-H tumors are typically characterized by high levels of TMB, neoantigen load and immune checkpoint expression [24–26], thus the above results suggest that FSTL3 expression level is closely related to microsatellite instability. Further analysis revealed that the expression level of FSTL3 was significantly higher in MSI-H tumors (Fig. 1E). Moreover, the proportion of MSI-H tumors was significantly higher in the group with high FSTL3 expression compared to the group with low FSTL3 expression (Fig. 1F). Receiver operating characteristic (ROC) analysis showed that the FSTL3 expression level had a good predictive effect on the microsatellite instability (Fig. 1G).

The cancer-immunity cycle (CIC) is referred to as a chain of occurrences required for the immune response to restrict tumor progression. Tumors can bypass immune surveillance when one or more CIC steps are compromised [27, 28]. In the group with high FSTL3 expression, macrophage recruitment and inflammatory immune cell infiltration activity were noticeably enhanced, while CD8^+^ T cell recruitment and cancer cell killing activity were reduced (Supplementary Fig. S3C). These results suggest that CRC with high FSTL3 expression is characterized by high levels of TMB, neoantigen load and microsatellite instability, and is closely associated with immune evasion.

### Hypoxia induces up-regulation of FSTL3 via HIF-1α in CRC cells

**Fig. 2:**
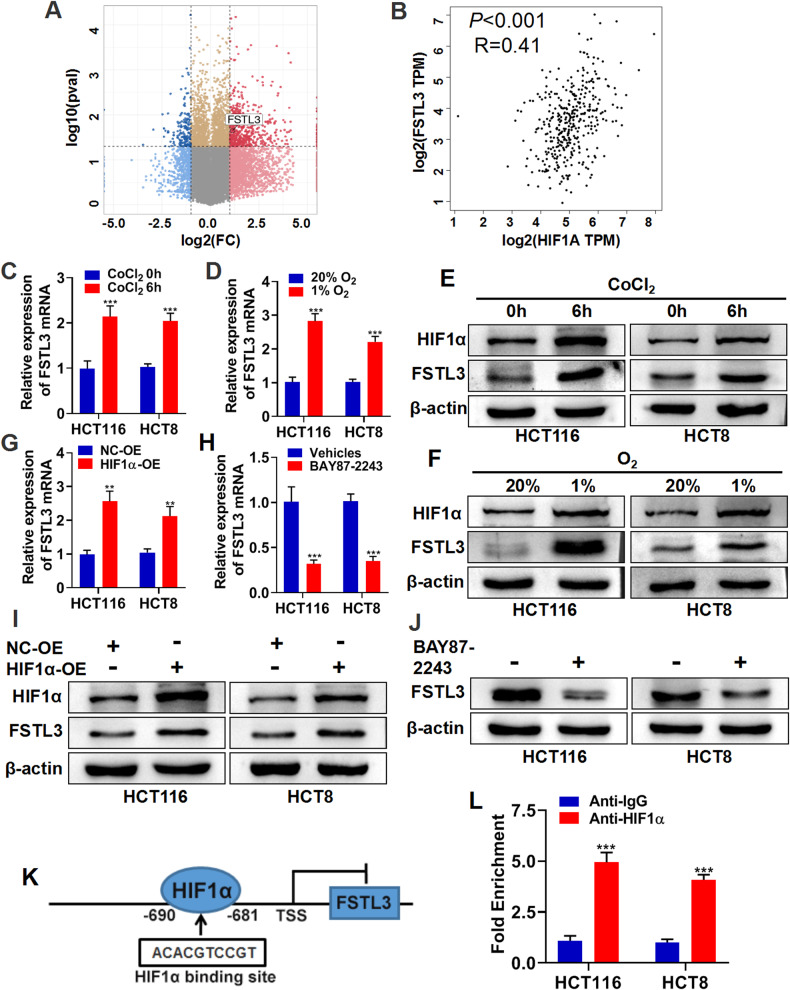
Hypoxia induces up-regulation of FSTL3 via HIF1α in in CRC cells. **A** Volcano plot showing the differentially expressed genes between RKO cells (human CRC cell line) with and without hypoxia treatment from GSE145108 dataset. **B** The correlation between the mRNA levels of HIF1α and FSTL3 in the TCGA CRC cohort. qRT-PCR was performed to detect FSTL3 mRNA in HCT116 and HCT8 cells after 100 μM CoCl_2_ treatment for 6 h (**C**) and incubation in hypoxic incubator (1% O_2_) for 24 h (**D**). Western blotting was performed to detect HIF1α and FSTL3 protein levels in HCT116 and HCT8 cells after CoCl_2_ treatment (**E**) and incubation in hypoxic incubator (**F**). The FSTL3 mRNA expression in HCT116 and HCT8 cells after overexpression of HIF1α via transfected plasmids examined (**G**) and administration of HIF1α inhibitor (BAY87-2243, 10 μM) (**H**) detected by qRT-PCR. The protein levels of HIF1α and FSTL3 in HCT116 and HCT8 cells after overexpression of HIF1α (**I**) and the administration of BAY87-2243 (**J**) examined by western blotting. **K** The potential binding sites of HIF1α to the FSTL3 promoter region predicted by the Jaspar2022 database. **L** ChIP and qRT-PCR were performed to detect the enrichment of HIF1α on the FSTL3 promoter. Data are shown as mean ± SD. **P < 0.01, ***P < 0.001.

Hypoxia is known as the predominant phenotype in solid tumors, and the hypoxia mediated by hypoxia-inducible factors (HIFs) is suspected to cause a number of genetic alterations in TME [29, 30]. Therefore, the effect of hypoxia on FSTL3 expression in CRC microenvironment was explored. By analyzing the differentially expressed genes in GSE145108, it was found out that the level of FSTL3 expression was significantly up-regulated in hypoxia-treated RKO cells (human CRC cell line) (Fig. 2A). Furthermore, there was a positive correlation observed between the mRNA expression of FSTL3 and hypoxia inducible factor-1alpha (HIF1α) in the TCGA CRC cohort (Fig. 2B). It was also discovered that the expression of FSTL3 mRNA and protein in HCT116 and HCT8 cells was substantially increased due to both hypoxic settings in medium enriched with CoCl_2_ and in hypoxic incubator (1% O_2_) constructions (Fig. 2C–F). By overexpressing HIF1α via plasmid and dampening HIF1α through HIF1α inhibitor (BAY87-2243), it was discovered that HIF1α played a role in regulating FSTL3 expression in HCT116 and HCT8 cells (Fig. 2G–J). Jaspar2022 database was used to investigate the potential HIF1α binding sites in the FSTL3 promoter region (Fig. 2K). According to the results of ChIP and qRT-PCR conducted on HCT116 and HCT8 cells, the promoter region of FSTL3 was enriched in the immunoprecipitate generated with anti-HIF1α antibody (Fig. 2L).

### FSTL3 positively regulates PDL1 expression in CRC cells

To elucidate the biological functions and mechanisms of FSTL3 in CRC development and immune evasion, lentivirus-mediated shFSTL3 and FSTL3^Flag^ cDNA were delivered to HCT116 and HCT8 cells, so as to establish steady FSTL3-KD and FSTL3-OE cells. The effectiveness of the knockdown and the overexpression of FSTL3 in HCT116 and HCT8 cells were confirmed by means of qRT-PCR and western blotting (Supplementary Fig. S4A, B). According to the results of the colony formation and EdU experiment, FSTL3 downregulation inhibited CRC cells proliferation whereas overexpression facilitated CRC cells growth (Supplementary Fig. S4C, D).

**Fig. 3:**
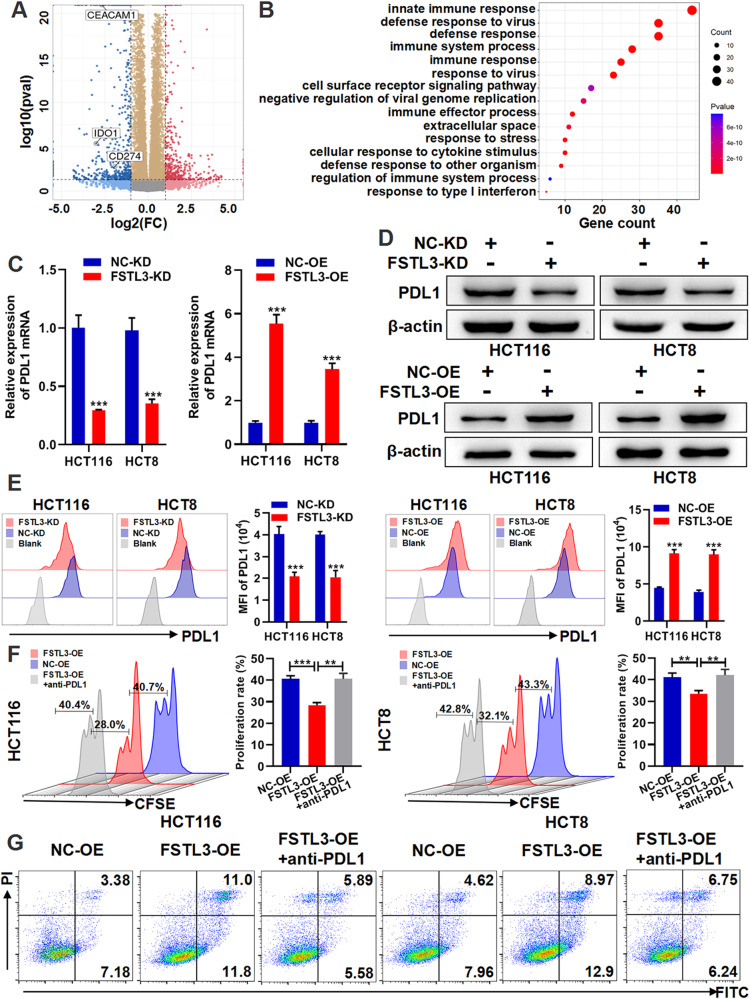
FSTL3 positively regulates PDL1 expression in CRC cells. **A** Volcano plot depicting differentially expressed genes in FSTL3 knockdown HCT116 cells. The immune checkpoint PDL1 (CD274), IDO1 and CEACAM1, which were down-regulated in expression, and marked by boxes. **B** GO analysis of the differentially expressed genes in FSTL3 knockdown HCT116 cells. The expression of PDL1 in FSTL3 knockdown and overexpressed CRC cells was detected by qRT-PCR (**C**), western blotting (**D**) and flow cytometry (**E**). **F** CFSE staining analysis indicating the proliferation ratio of CD8^+^ T cells after co-culture with FSTL3-OE cells in the absence or presence of an anti-PDL1 neutralizing antibody by flow cytometry. **G** Flow cytometry apoptosis analysis showing the apoptotic percentage of CD8^+^ T cells after co-culture with FSTL3-OE cells in the absence or presence of an anti-PDL1 neutralizing antibody. Data are shown as mean ± SD. **P < 0.01, ***P < 0.001.

Next, we analyzed the transcription profiles of HCT116 cells with and without FSTL3 knockdown by RNA-sequencing. It was discovered that FSTL3 knockdown suppressed the expression of immune checkpoint PDL1, IDO1 and CEACAM1 (Fig. 3A). Gene ontology analysis revealed that the biological processes which differentially expressed genes predominantly enriched in were pertinent to the immune response (Fig. 3B). The abnormal hyperexpression of PDL1 and IDO1 of cancer cells plays a crucial role in immune tolerance and immune evasion [31, 32]. The immunotherapy targeting PDL1/PD1 has demonstrated its massive potential in terms of cancer treatment [33]. The application of IDO1 inhibitors also represents a promising immunotherapeutic strategy [34]. Hence, PDL1 and IDO1 were taken as downstream targets for follow-up experiments. Firstly, we opted for PDL1 to analyze. The regulator role of FSTL3 on PDL1 was verified by qRT-PCR, western blotting and flow cytometry in combination (Fig. 3C, E). The binding of PDL1 on cancer cells to PD1 on CD8^+^ T cells induces apoptosis and inhibits the proliferation of CD8^+^ T cells, leading to immune evasion [35]. Thus, we constructed a co-culture model of FSTL3-OE CRC cells with CD8^+^ T cells to explore the effect of FSTL3 in cancer cells on CD8^+^ T cells. According to CFSE staining analysis, FSTL3-OE cells inhibited the proliferation of CD8^+^ T cells, which could be attenuated by anti-PDL1 antibody (Fig. 3F). As revealed by flow cytometry apoptosis analysis, FSTL3-OE cells triggered the apoptosis of CD8^+^ T cells, while the anti-PDL1 antibody could weaken this change (Fig. 3G). Therefore, it is suggested that FSTL3 positively regulates PDL1 expression in CRC cells and promotes immune escape.

### FSTL3 binds directly to c-Myc and enhances the stability of c-Myc in CRC cells

**Fig. 4:**
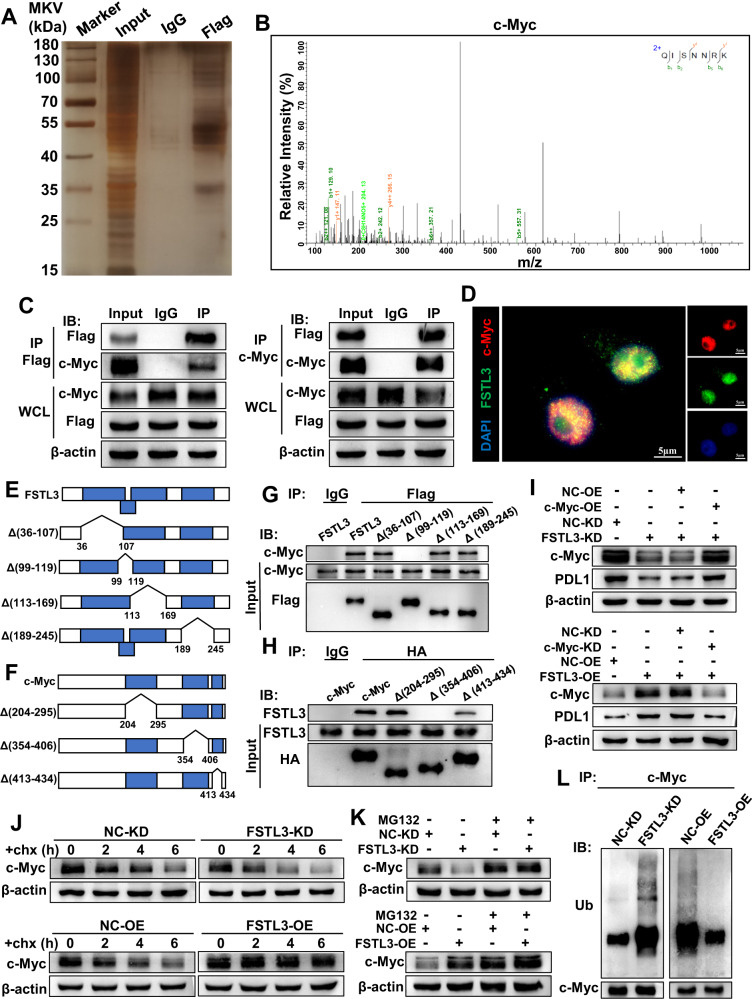
FSTL3 binds directly to c-Myc and enhances the stability of c-Myc in CRC cells. **A** Immunoprecipitate captured by anti-Flag antibody from FSTL3-OE HCT116 cells was separated by SDS-PAGE gel followed by silver staining. **B** The peptide sequences of the c-Myc protein as detected in the immunoprecipitate complex by mass spectrometry. **C** Western blotting analysis of immunoprecipitate complexes captured from FSTL3-OE HCT116 cells against anti-Flag or anti-c-Myc antibody. **D** Immunofluorescence observation revealed that FSTL3 co-localized with c-Myc in the nucleus of HCT116 cells. **E** Schematic diagram of full-length FSTL3 and its various deletion mutants. **F** Schematic diagram of full-length c-Myc and its various deletion mutants. **G** The plasmids of Flag-FSTL3 full-length and specific Flag-FSTL3-depletion fragments were transfected into HCT116 cells, followed by immunoprecipitation using anti-Flag antibody and western blotting to assess the specific region of FSTL3 binding to c-Myc. **H** The plasmids of HA-c-Myc full-length and specific HA-c-Myc-depletion fragments were transfected into HCT116 cells, followed by immunoprecipitation using anti-HA antibody and western blotting to assess the specific region of c-Myc binding to FSTL3. **I** Western blotting analysis of c-Myc and PDL1 expression in HCT116 cells transfected with FSTL3 shRNA or together with c-Myc over-expression plasmid, and in HCT116 cells transfected with FSTL3 over-expression lentivirus or together with c-Myc siRNA. **J** Stability analysis of c-Myc protein in FSTL3-KD and FSTL3-OE HCT116 cells treated with 40 μM cycloheximide (chx) for indicated times. **K** Western blotting was used to detect the expression of c-Myc protein in FSTL3-KD and FSTL3-OE HCT116 cells treated with 10 μM MG132. **L** Ubiquitination assay of c-Myc in FSTL3-KD and FSTL3-OE HCT116 cells.

To elucidate the mechanism of the PDL1 regulated by FSTL3 in CRC cells, immunoprecipitation and LC–MS/MS were performed by using anti-Flag antibody on the Flag-tagged FSTL3-OE HCT116 cell lysates. Among the immunoprecipitate identified by MS, the focus of attention was MYC proto-oncogene, bHLH transcription factor (c-Myc) (Fig. 4A, B), which can regulate the transcriptional expression of PDL1 by binding to the PDL1 promoter and act as an essential motor of tumor growth and immune evasion [36]. Anti-Flag or anti-c-Myc antibody was applied to FSTL3-OE HCT116 cell lysates for immunoprecipitation, which was followed by the western blotting of immunoprecipitate complexes. According to the results, FSTL3 can bind to c-Myc in CRC cells (Fig. 4C). Additionally, IF analysis revealed that FSTL3 was co-localized with c-Myc in the nucleus of HCT116 cells (Fig. 4D). To briefly describe the structural domains in which FSTL3 and c-Myc interact, the plasmids of specific Flag-FSTL3-depletion or HA-c-Myc-depletion fragments were constructed to transfect HCT116 cells (Fig. 4E, F). Then, the immuoprecipitation of transfected cell lysates was performed by using anti-Flag or anti-HA antibody. As suggested by the results, the FSTL3 protein containing the (99–119aa) fragment could bind to the c-Myc protein, and the c-Myc protein containing the (354–406aa) fragment could bind to the FSTL3 protein (Fig. 4G, H). In addition, it was discovered that the levels of c-Myc protein were substantially downregulated in FSTL3-KD HCT116 cells but significantly upregulated in FSTL3-OE HCT116 cells, despite no significant change to the mRNA expression of c-Myc. Meanwhile, c-Myc remodeling mediated the level of PDL1 expression in FSTL3-KD or FSTL3-OE HCT116 cells (Fig. 4I and Supplementary Fig. S5A–D). The impact of FSTL3 on the stability of the c-Myc protein was evaluated by using cycloheximide (chx). Compared with the control cells, FSTL3 knockdown was accompanied with a reduction in the stability of c-Myc protein, while FSTL3 overexpression was closely associated with the improved stability of c-Myc protein (Fig. 4J). With the administration of MG132 to conditioned medium, the impact of FSTL3 down- or up-regulation on c-Myc expression levels in HCT116 cells was significantly mitigated (Fig. 4K). We subsequently proceeded with ubiquitination assays. The level of ubiquitination of c-Myc was significantly raised in FSTL3-KD HCT116 cells. In contrast, the over-expression of FSTL3 enhanced the deubiquitination of c-Myc (Fig. 4L). To sum up, these results illustrate that FSTL3 protein can interact with c-Myc protein directly and enhance the stability of c-Myc protein in CRC cells.

### Hyper-expressed FSTL3 in CRC cells enhances the c-Myc/IDO1 pathway and induces Tregs

**Fig. 5:**
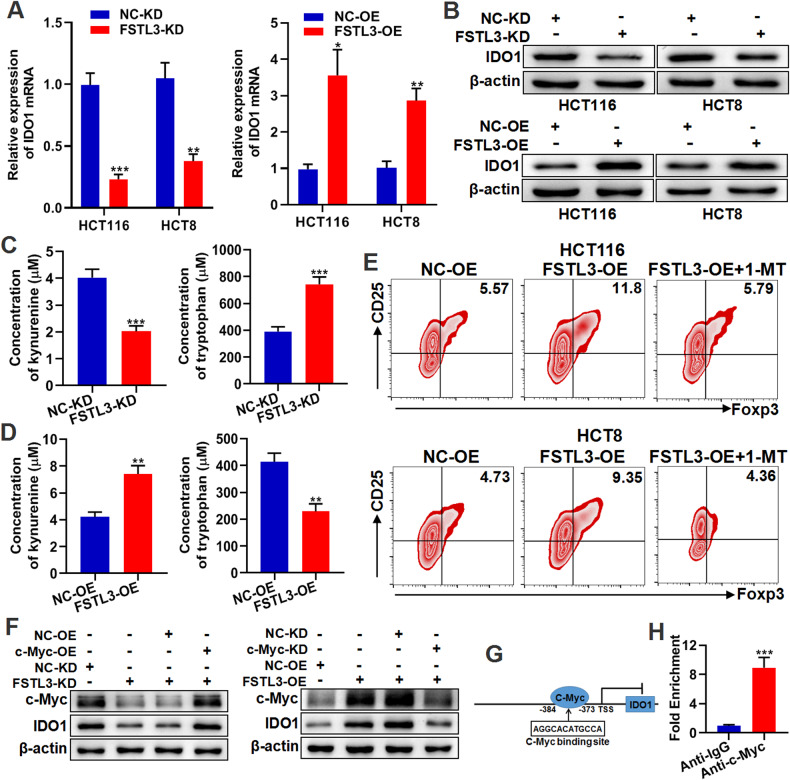
Hyper-expressed FSTL3 in CRC cells enhances the c-Myc/IDO1 pathway and induces Tregs. The expression of IDO1 in FSTL3-KD and FSTL3-OE CRC cells was detected by qRT-PCR (**A**) and western blotting (**B**). **C** Concentration of kynurenine and tryptophan in the cell supernatants of FSTL3-KD MC38 cells detected by ELISA. **D** Concentration of kynurenine and tryptophan in the cell supernatants of FSTL3-OE MC38 cells detected by ELISA. **E** The proportion of Tregs in CD4^+^ T cells after co-culture with FSTL3-OE CRC cells in the absence or presence of 1-MT. **F** Western blotting analysis of c-Myc and IDO1 expression in CRC cells transfected with FSTL3 shRNA or together with c-Myc over-expression plasmid, and in CRC cells transfected with FSTL3 over-expression lentivirus or together with c-Myc siRNA. **G** The potential binding sites of c-Myc to the IDO1 promoter region predicted by the Jaspar2022 database. **H** ChIP and qRT-PCR were performed to detect the enrichment of c-Myc on the IDO1 promoter. Data are shown as mean ± SD. **P < 0.01, ***P < 0.001.

Apart from PDL1 mRNA, IDO1 mRNA was also significantly down-regulated in FSTL3-KD CRC cells according to anterior RNA-sequencing. Here we investigated IDO1 as a target gene. Then, the results of qRT-PCR and western blotting were obtained to verify the regulator role of FSTL3 on IDO1 (Fig. 5A, B). IDO1 is a type of enzyme that plays a vital role in tryptophan (Trp) cleavage and kynurenine (Kyn) synthesis [37]. According to the Kyn/Try detection assays, the occurrence of FSTL3 knockdown in MC38 cells reduced Kyn levels but increased Try levels in cell supernatants (Supplementary Fig. S6A and Fig. 5C). Elevated Kyn concentrations and declined Try concentrations were detected in supernatants of FSTL3-OE MC38 cells (Supplementary Fig. S6A and Fig. 5D). After the co-culturing of CD4^+^ T cells with NC-OE and FSTL3-OE CRC cells, the proportion of Tregs (CD25^+^ Foxp3^+^ T cells) among CD4^+^ T cells increased in the FSTL3-OE group, while IDO1 inhibitor (1-MT) could weaken this change (Fig. 5E). To investigate the mechanism of FSTL3 regulation of IDO1, c-Myc was overexpressed in FSTL3-KD CRC cells and knocked down in FSTL3-OE CRC cells. According to the results of western blotting, c-Myc can modulate the expression of IDO1 in HCT116 cells (Fig. 5F). After a potential binding site was identified for the promoter region of IDO1 to c-Myc based on the Jaspar2022 database, the site was validated by ChIP and qRT-PCR (Fig. 5G, H). To sum up, FSTL3 in CRC cells can enhance the c-Myc/IDO1 pathway and induce Tregs by regulating Kyn/Try metabolism.

### Knockdown of FSTL3 in CRC fosters the establishment of anti-tumor immune microenvironment

**Fig. 6:**
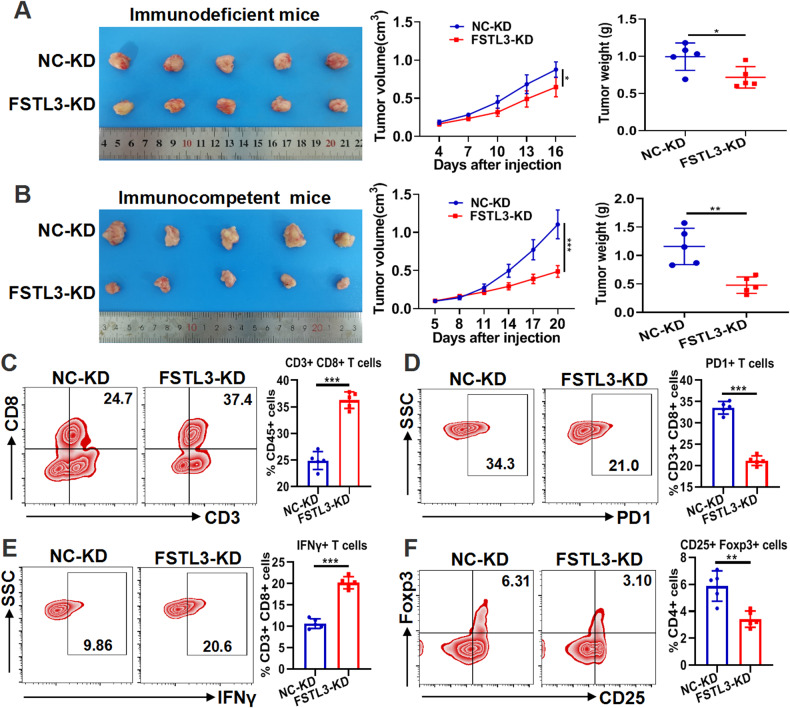
Knockdown of FSTL3 in MC38 cells fosters the establishment of anti-tumor immune microenvironment in CRC. **A** Immunodeficient mouse (BABL/c-nude mouse) tumor models were constructed by subcutaneous implantation of MC38 cells with FSTL3-KD or NC-KD. Tumor images, growth curves and weight were obtained at day 16 after dissection. **B** Immunocompetent mouse (C57BL/6J mouse) tumor models were constructed by subcutaneous implantation of FSTL3-KD or NC-KD MC38 cells (FSTL3-KD group or NC-KD group). Tumor images, growth curves and weight were obtained at day 20 after dissection. **C** Flow cytometric analysis of the infiltrated CD8^+^ T cells in tumor tissues of C57BL/6J mice in each group. Flow cytometric analysis of PD1 (**D**) and IFNγ (**E**) in CD8^+^ T cells in tumor tissues of C57BL/6J mice in each group. **F** Flow cytometric analysis of Tregs (CD25^+^ Foxp3^+^ T cells) in tumor tissues of C57BL/6J mice in each group. Data are shown as mean ± SD. *P < 0.05, **P < 0.01, ***P < 0.001.

To further reveal the effect of FSTL3 on tumor growth and tumor immunity of CRC, immunocompetent and immunodeficient mouse tumor models were constructed by subcutaneously implanting FSTL3-KD or NC-KD MC38 cells, which were transfected with lentivirus-mediated shFSTL3 or shNC (Supplementary Fig. S6A). It was observed that FSTL3 knockdown inhibited the growth of subcutaneous tumors in both immunodeficient and immunocompetent mice, and that the inhibitory effect was more significant in immunocompetent mice (Fig. 6A, B). It suggests the potential of FSTL3 in cancer cells to affect tumor growth through tumor immunity. Subsequently, the phenotypic characteristics of immune cells in the tumors of immunocompetent mice were analyzed by flow cytometry. It was found out that the FSTL3 knockdown in MC38 cells resulted in an ascending proportion of CD8^+^ T cells in the tumors, accompanied by decreased expression of the inhibitory receptor PD1 and heightened production of the effector cytokine IFNγ in CD8^+^ T cells (Fig. 6C–E). Besides, FSTL3 knockdown suppressed Tregs infiltration in tumors (Fig. 6F). The above results imply that the down-regulation of FSTL3 expression can suppress tumor growth by enhancing the anti-tumor effects of CD8^+^ T cells and repressing the infiltration of Tregs in CRC. Previous studies have reported FSTL3 is closely associated with macrophages in CRC [15, 16], and immunohistochemistry results showed that downregulation of FSTL3 suppressed the expression of F4/80, CD163, and CD206 in mouse tumors (Supplementary Fig. S6B), suggesting that FSTL3 promotes the infiltration and polarization of TAMs in CRC.

### FSTL3 serves as a potential biomarker of resistance to ICB therapy

**Fig. 7:**
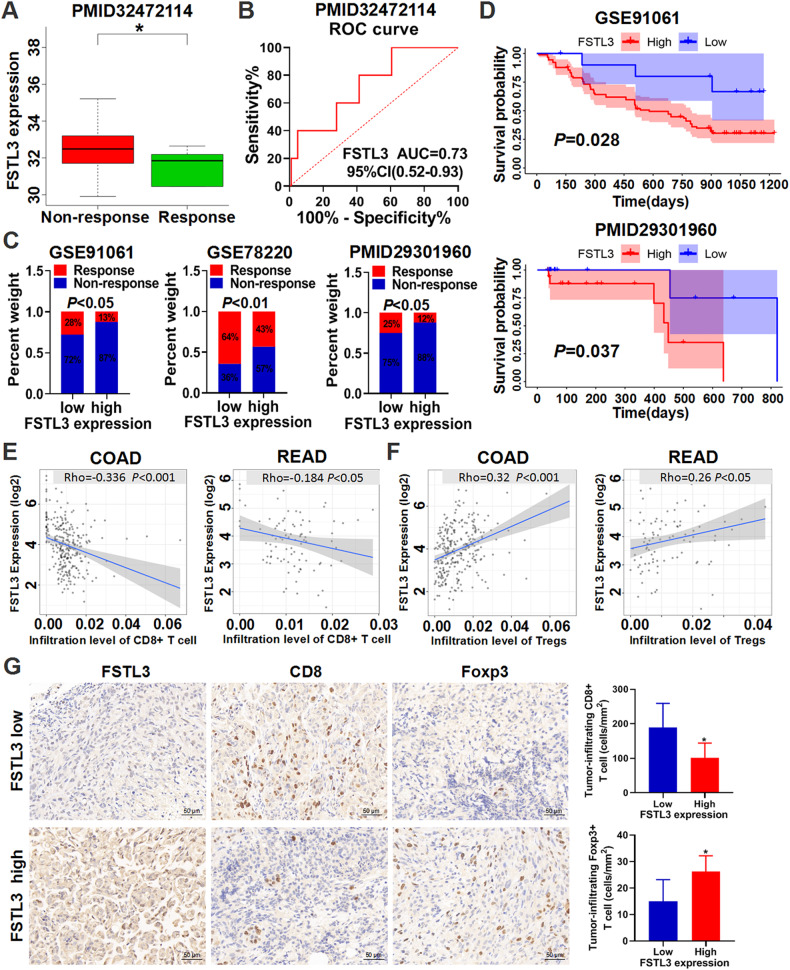
FSTL3 serves as a potential biomarker of resistance to ICB therapy. **A** Box plots showing the expression of FSTL3 in the response group and non-response group. **B** ROC curve showing the predictive efficiency of FSTL3 expression for ICB therapy response. **C** The response rate of ICB therapy in high versus low FSTL3 expression subgroups in the cohorts GSE91061, GSE78220, and PMID29301960. **D** Kaplan–Meier analysis of the relationship between FSTL3 expression level and overall survival of patients from the GSE91061 and PMID29301960 cohorts. **E** The correlation between FSTL3 expression and CD8^+^ T cells infiltration in colon and rectal adenocarcinoma tissues. **F** The correlation between FSTL3 expression and Tregs infiltration in colon and rectal adenocarcinoma tissues. **G** Representative images of FSTL3, CD8 and Foxp3 staining in high versus low FSTL3 expression CRC tissues. Data are shown as mean ± SD. *P < 0.05.

Based on the finding that FSTL3 downregulation improved CD8^+^ T cells antitumor activity and reduced Tregs infiltration in mice tumors, the role of FSTL3 expression in the response to ICB therapy was further investigated. We collected transcriptomic profiles and clinical information from ICB therapy cohort PMID32472114. In this cohort, FSTL3 expression level in the non-response group was significantly higher than that in the response group (Fig. 7A). To assess the predictive efficiency of FSTL3 expression levels on ICB therapy response, we generated a ROC curve. The area under the ROC curve was 0.73, indicating a high predictive value (Fig. 7B). We additionally collected transcriptome profiles and clinical data from the ICB therapy cohorts GSE91061, GSE78220 and PMID29301960 for analysis, and found that the response rate was lower in the cases with high FSTL3 expression compared to those with low FSTL3 expression (Fig. 7C). Moreover, in the GSE91061 and PMID29301960 cohorts, the patients with high FSTL3 expression survived a substantially shorter period (Fig. 7D). Additionally, we found that FSTL3 expression levels were negatively correlated with CD8^+^ T cell infiltration levels and positively correlated with Tregs infiltration levels in colon and rectal adenocarcinoma tissues (Fig. 7E, F). IHC results demonstrated that CD8^+^ T cells infiltration levels were lower and Tregs infiltration levels were higher in CRC tissues with high FSTL3 expression compared to those with low FSTL3 expression (Fig. 7G). Therefore, it was speculated that resistance to ICB therapy occurred in the cases with high FSTL3 expression, which might be associated with dysregulated CD8^+^ T cells and Tregs.

### Hyper-expressed FSTL3 resists the antitumor efficacy of anti-PD1 therapy in vivo

**Fig. 8:**
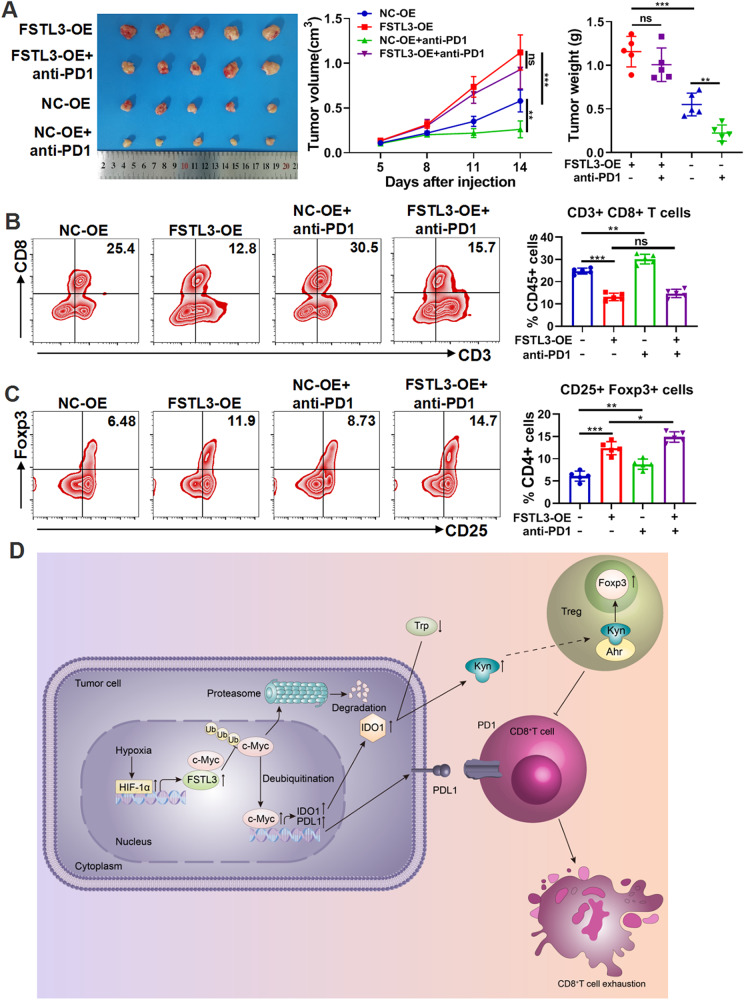
Hyper-expressed FSTL3 resists the antitumor efficacy of anti-PD1 therapy in vivo. The mice were injected subcutaneously with NC-OE or FSTL3-OE MC38 cells to establish tumor models, and then treated with anti-PD1 antibody. **A** Tumor images, growth curves and weight were obtained at day 14 after dissection. **B** Flow cytometry analysis of CD8^+^ T cells in tumor tissues of C57BL/6 J mice in each group. **C** Flow cytometry analysis of Tregs in tumor tissues of C57BL/6 J mice in each group. **D** Schematic diagram of the concrete function and mechanism of FSTL3 in the CRC microenvironment. Data are shown as mean ± SD. *P < 0.05, **P < 0.01, ***P < 0.001, ns non-significant.

To further evaluate the impact of high FSTL3 expression on ICB therapy, mouse subcutaneous tumor models were constructed with NC-OE and FSTL3-OE MC38 cells, which were transfected with lentivirus-mediated control cDNA and FSTL3^Flag^ cDNA (Supplementary Fig. S6A), followed by anti-PD1 therapy. In comparison with the NC-OE+anti-PD1 group, anti-PD1 therapy failed to achieve a significant anti-tumor effect in the FSTL3-OE+anti-PD1 group, as shown in Fig. 8A. According to flow cytometry analysis, anti-PD1 therapy led to a significant increase in the proportion of CD8^+^ T cells in the tumors of NC-OE+anti-PD1 group in comparison with the NC-OE group, while there was no significant increase observed in the tumors of FSTL3-OE+anti-PD1 group in comparison with the FSTL3-OE group (Fig. 8B). Meanwhile, anti-PD1 therapy made no significant difference to the expression of PD1 or IFNγ in CD8^+^ T cells in the tumors of FSTL3-OE+anti-PD1 group in comparison with the FSTL3-OE group (Supplementary Fig. S7A, B). In addition, the infiltration of Tregs was substantially enhanced in the tumors of NC-OE + anti-PD1 group and FSTL3-OE+anti-PD1 group, respectively, compared with the group without anti-PD1 treatment (Fig. 8C). To further establish whether the high expression of FSTL3 made ICB resistance dependent on the increase of IDO1, mouse subcutaneous tumor models were constructed by using FSTL3-OE MC38 cells and a combination therapy of anti-PD1 antibody and 1-MT was administered. As a result, 1-MT inhibited tumor growth, and the inhibitory effect became more significant when it was combined with anti-PD1 antibody (Supplementary Fig. S8A–C). Additionally, it was observed that the HIF1α inhibitor (BAY87-2243) inhibited the growth of subcutaneous tumors constructed by FSTL3-OE MC38 cells, suggesting the potentially value of BAY87-2243 for CRC patients with high FSTL3 expression (Supplementary Fig. S8D–F). In summary, the high expression of FSTL3 can lead to anti-PD1 therapy resistance, and the combination with IDO1 inhibitor can sensitize the therapy for a better anti-tumor effect.

## Discussion

In the present study, it is found out that FSTL3 is not only hyper-expressed in cancer cells of CRC but also correlated with immune evasion. In cancer cells, FSTL3 prevented the ubiquitination and degradation of c-Myc through interaction with c-Myc, triggered the transcriptional expression of downstream PDL1 and IDO1, and thus blocked the anti-tumor effect of CD8^+^ T cells and enhanced the infiltration of Tregs (Fig. 8D). Furthermore, the hyper-expression of FSTL3 resulted in the resistance to anti-PD1 therapy, which can be reversed by the combination with IDO1 inhibitor.

PDL1 is extensively presented on T cells, B cells, dendritic cells, macrophages and various human cancer cells [38]. As an immune checkpoint, PDL1 can not only induce CD8^+^ T cells depletion and apoptosis but also suppress CD8^+^ T cells proliferation and cytotoxicity by binding to PD1 on the surface of CD8^+^ T cells [35]. In this study, it was revealed through cellular and animal experiments that the FSTL3 in CRC cells could upregulate PDL1 expression and mitigate the antitumor effect of CD8^+^ T cells to promote immune evasion. In TME, PDL1 expression is modulated in different ways, including at transcriptional, post-transcriptional and post-translational levels. The regulators at the transcriptional level include interferon regulatory factor 1 (IRF-1), nuclear factor kappa B subunit (NF-κB) pathway, mitogen-activated protein kinase (MAPK) pathway and c-Myc, etc [39]. As revealed in our experiments, FSTL3 concentrated in the nucleus in CRC, implicating a specific intracellular function for this protein. As indicated by Forissier et al., nucleus FSTL3 plays a role in transcriptional regulation through interaction with MLLT10 [40]. As a type of FSTL3 homologue, follistatin like 1 (FSTL1) was found as capable to bind to pyruvate kinase M1/2 (PKM2) and reduce the ubiquitination and instability of PKM2 [41]. We further found out that the FSTL3 in the nucleus bound to c-Myc via the (99–119aa) fragment, which exhibited FSTL1 characteristics, and that FSTL3 can get involved in transcriptional regulation by modulating the ubiquitination and stability of c-Myc.

The US Food and Drug Administration (FDA) has approved the indication of ICB for MSI-H solid tumors, including CRC [42]. The frontline use of ICB has been supported by phase III evidence in the MSI-H CRC [43, 44]. However, the objective remission rate of ICB in MSI-H CRC was only 31.7–62.5%, which means that a significant proportion of them are resistant to ICB therapy [45], hence additional biomarkers are required to guide a more personalized treatment. In this study, we found that CRC with high FSTL3 expression is characterized by high levels of TMB, neoantigen load and microsatellite instability. Moreover, FSTL3 can be used as a barometer of MSI-H with good predictive power. Therefore, it is suggested that ICB may be suitable for CRC with high FSTL3 expression. However, public database mining and experiments showed that high-expressing FSTL3 patients with dysregulated CD8^+^ T cells and Tregs were resistant to ICB therapy, which can be alleviated by the combination of IDO1 inhibitor.

As a major barrier to host antitumor activity, the immunosuppressive effect can result in the inferior response to immunotherapy in CRC patients [2, 46]. Among the most enriched immunosuppressive cells in TME, Tregs can lead to CD8^+^ T cells dysfunction, immune evasion, and immunotherapy resistance through intercellular contacts as well as the secretion of cytokines and metabolites [47–49]. We observed that the hyper-expression of FSTL3 could active c-Myc/IDO1 pathway and promote Trp depletion and Kyn accumulation. Both the activation of IDO1 and the level of Kyn/Trp are suspected as responsible for CD8^+^ T cell depletion and immunotherapy resistance [50, 51]. Kyn, a metabolite synthesized by IDO1, can bind to the aryl hydrocarbon receptor and activates it, thereby facilitating Tregs initiation and impairing the function of CD8^+^ T cells [52, 53]. In this study, it was found that overexpression of FSTL3 induced Tregs infiltration and resistance to ICB therapy in mouse CRC tumors. Treg has been identified as a critical element in the resistance to ICB in multiple cancers, such as CRC, non-small cell lung cancer, breast cancer and glioblastomas [54–57]. Although the CRC patients with high FSTL3 expression may be indicated for ICB treatment due to the characteristics of MSI-H, their high levels of IDO1 expression and Tregs infiltration result in ICB resistance. It is implied that FSTL3 can guide a more personalized treatment for ICB-resistant MSI-H CRC patients as a biomarker.

To conclude, FSTL3 enhanced c-Myc-mediated transcriptional regulation to promote immune evasion in CRC. Additionally, the overexpression of FSTL3 can induce anti-PD1 therapy resistance in mice tumors, which can be mitigated with IDO1 inhibitor. Therefore, FSTL3 is considered as a potential biomarker of immunotherapeutic efficacy and a novel immunotherapeutic target in CRC patients, particularly in ICB-resistant MSI-H CRC patients.

### Supplementary information

Supplementary figures

Supplementary tables

Full and uncropped western blots

A reproducibility checklist

## Data Availability

The data obtained and/or analyzed during the current study were available from the corresponding authors in a reasonable request.
