# Supplementary material for: FSTL3 promotes tumor immune evasion and attenuates response to anti-PD1 therapy by stabilizing c-Myc in colorectal cancer
Source: Cell Death Dis. 2024 Feb 1;15(2):107. doi: 10.1038/s41419-024-06469-0 (PMC10834545; doi:10.1038/s41419-024-06469-0)
Supplement: Supplementary file 1 — Supplementary figures [file 41419_2024_6469_MOESM1_ESM.docx]

**Supplementary Figures**

**Supplementary Figure S1**

**
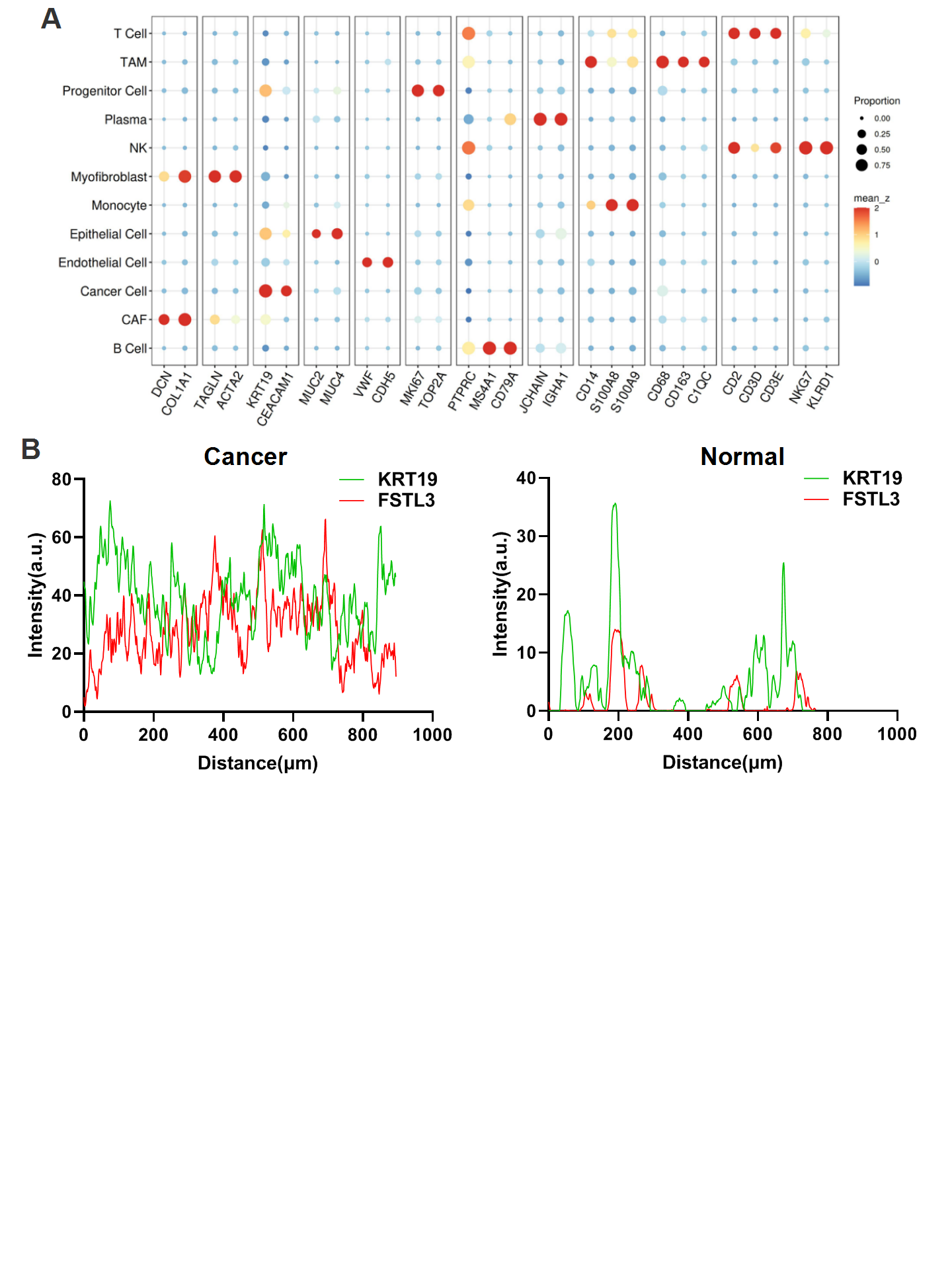
**

**Supplementary Figure S1. Supplement to Figure 1. (A)** Prominent cell type markers for the 12 cell clusters. **(B)** Quantitative assessment of FSTL3 (red) and KRT19 (green) expression in CRC tissues performed by ImageJ software.

**Supplementary Figure S2**

**
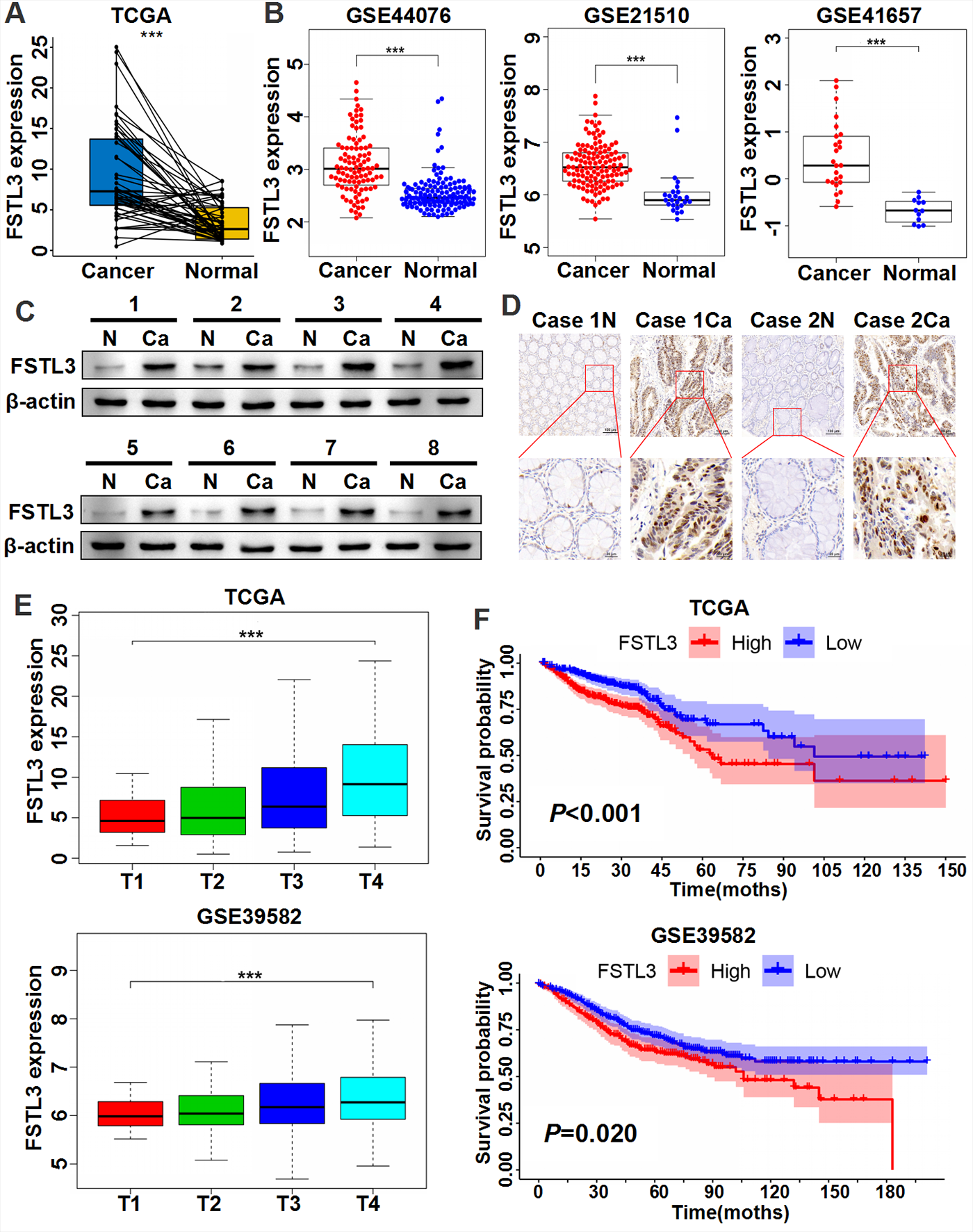
**

**Supplementary Figure S2. FSTL3 is highly expressed in CRC tissues and correlates with poor prognosis. (A)** The expression of FSTL3 in tumor tissues and matched normal tissues in the TCGA CRC cohort. **(B)** FSTL3 expression in CRC samples from the GSE44076, GSE21510 and GSE41657 datasets. **C** Western blotting was applied to detect the expression of FSTL3 protein levels in clinical CRC samples. **(D)** Immunohistochemistry analysis of FSTL3 protein expression in clinical CRC samples. **(E)** Expression of FSTL3 in different T-stage CRC samples from TCGA CRC cohort and GSE39582 dataset. **(F)** Kaplan-Meier analysis of the relationship between FSTL3 expression level and overall survival of CRC patients from the TCGA CRC cohort and GSE39582 dataset. Data are shown as mean ± SD. ****P* < 0.001.

**Supplementary Figure S3**

**
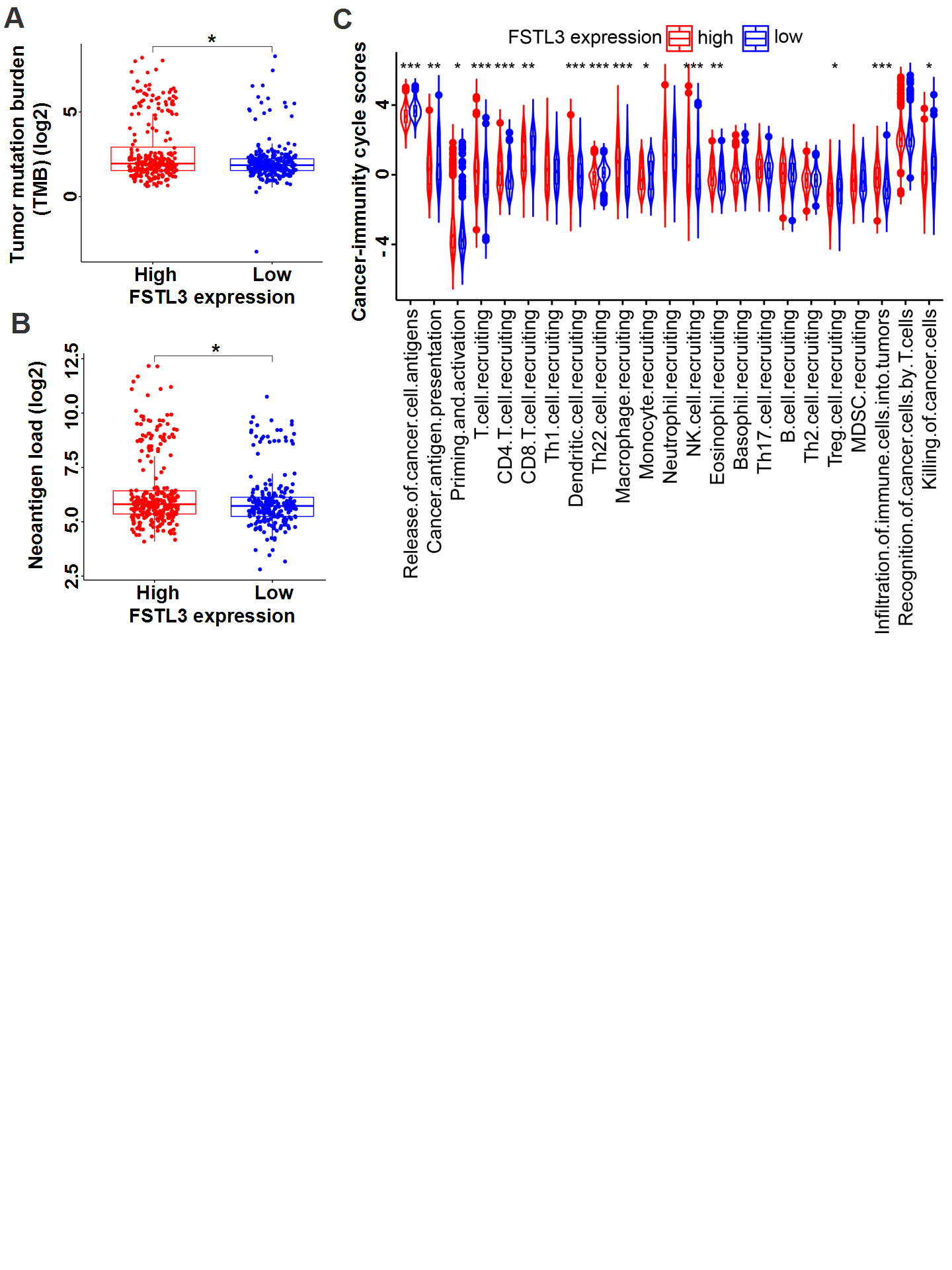
**

**Supplementary Figure S3. Correlations of tumor mutation burden (TMB), neoantigen load and cancer-immunity cycle with FSTL3 expression levels. (A)** TMB in high versus low FSTL3 expression subgroups. **(B)** Neoantigen load in high versus low FSTL3 expression subgroups. **(C)** Score differences between the various steps of the cancer-immunity cycle in the high- and low-FSTL3 groups. Data are shown as mean ± SD. **P* < 0.05, ***P* < 0.01, ****P* < 0.001.

**Supplementary Figure S4**

**
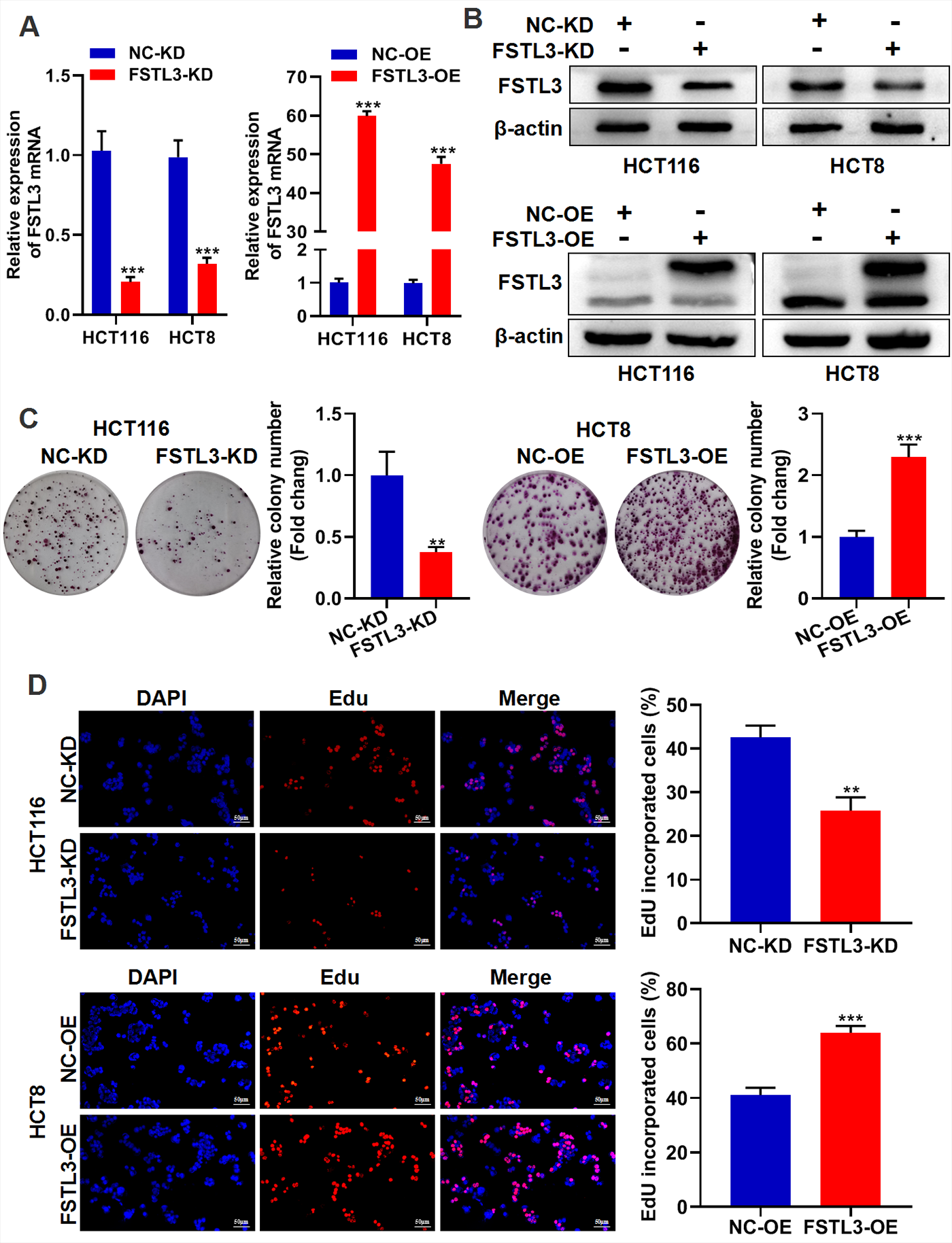
**

**Supplementary Figure S4. FSTL3 facilitates the proliferation of CRC cells. (A-B)** Lentivirus-mediated FSTL3-specific short hairpin RNA (shFSTL3) and FSTL3^Flag^ cDNA were transfected into HCT116 and HCT8 cells to establish steady FSTL3 knockdown (FSTL3-KD) and FSTL3 overexpressing (FSTL3-OE) cells. qRT-PCR (A) and western blotting (B) were performed to verify the transfection efficiency. **(C)** Colony formation was used to evaluate the effect of FSTL3 on the proliferation of CRC cells. **(D)** The proliferation viability of FSTL3-KD and FSTL3-OE cells was measured by EdU assay. Data are shown as mean ± SD. ***P* < 0.01, ****P* < 0.001.

**Supplementary Figure S****5**


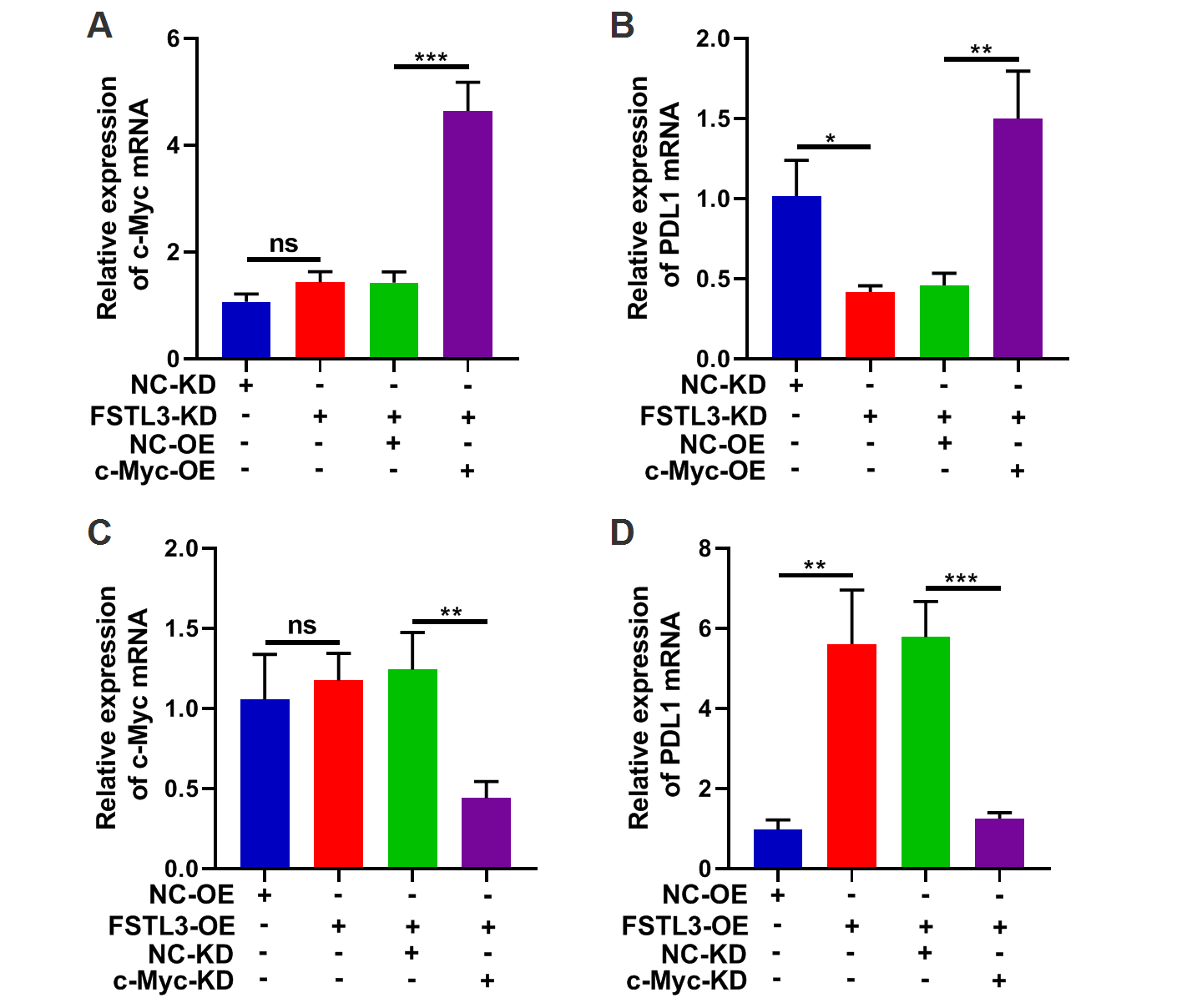


**Supplementary Figure S5. The mRNA expression of c-Myc and PDL1 in CRC cells with different treatments. (A-B)** The mRNA expression of c-Myc (A) and PDL1 (B) in HCT116 cells transfected with FSTL3 shRNA or together with c-Myc over-expression plasmid. **(C-D)** The mRNA expression of c-Myc (C) and PDL1 (D) in HCT116 cells transfected with FSTL3 over-expression lentivirus or together with c-Myc siRNA. Data are shown as mean ± SD. **P* < 0.05, ***P* < 0.01, ****P* < 0.001, ns, non-significant.

**Supplementary Figure S6**


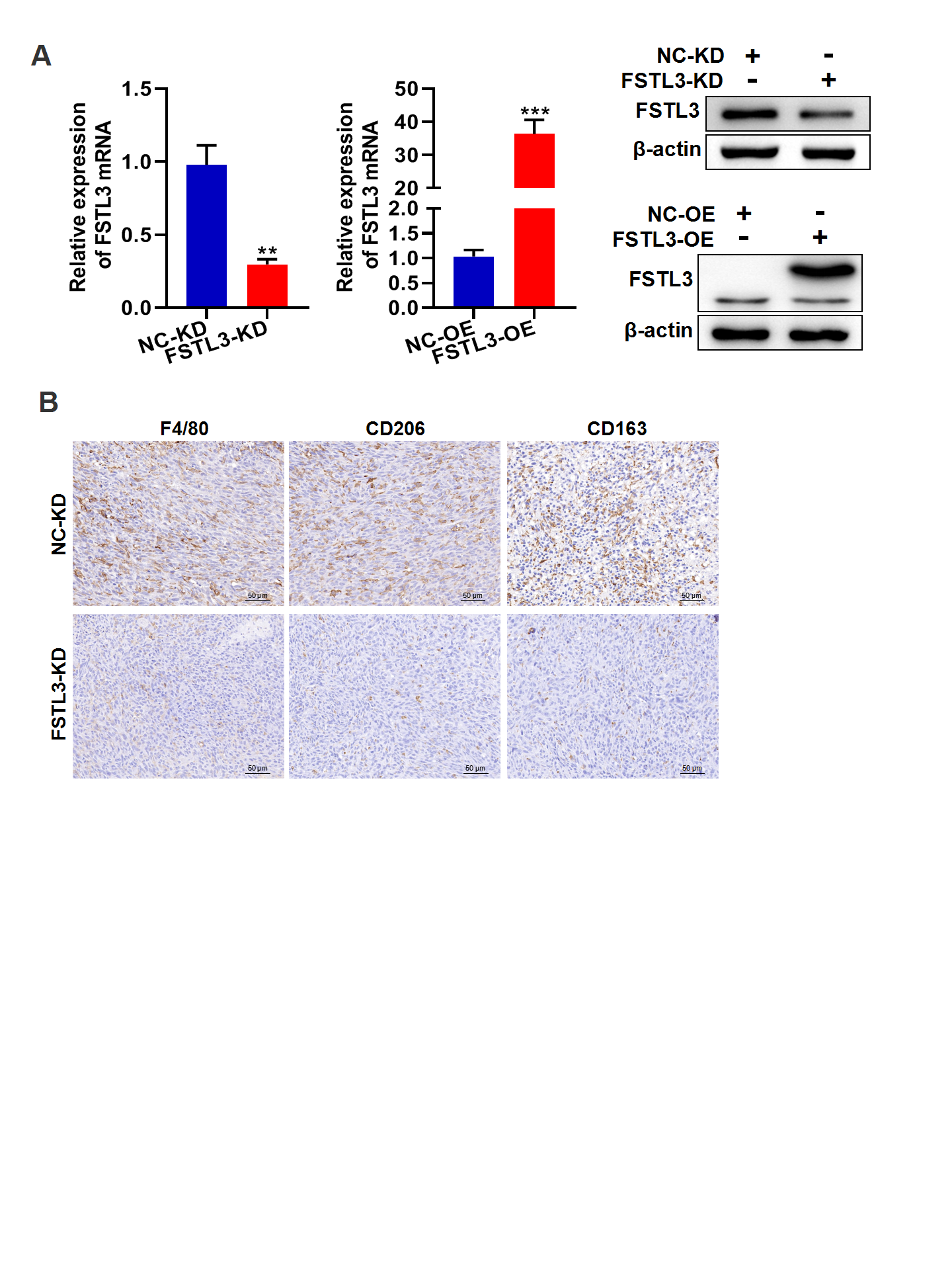


**Supplementary Figure S6.** **Knockdown of FSTL3 in MC38 cells inhibits macrophages infiltration in CRC. (A)** The mRNA and protein expression levels of FSTL3 in MC38 cells transfected with lentivirus-mediated shFSTL3 and FSTL3^Flag^ cDNA (FSTL3-KD and FSTL3-OE MC38 cells. **(B)** Immunohistochemical analysis of F4/80, CD206 and CD163 expression in mouse tumors established with NC-KD or FSTL3-KD MC38 cells. Data are shown as mean ± SD. ***P* < 0.01, ****P* < 0.001.

**Supplementary Figure S7**


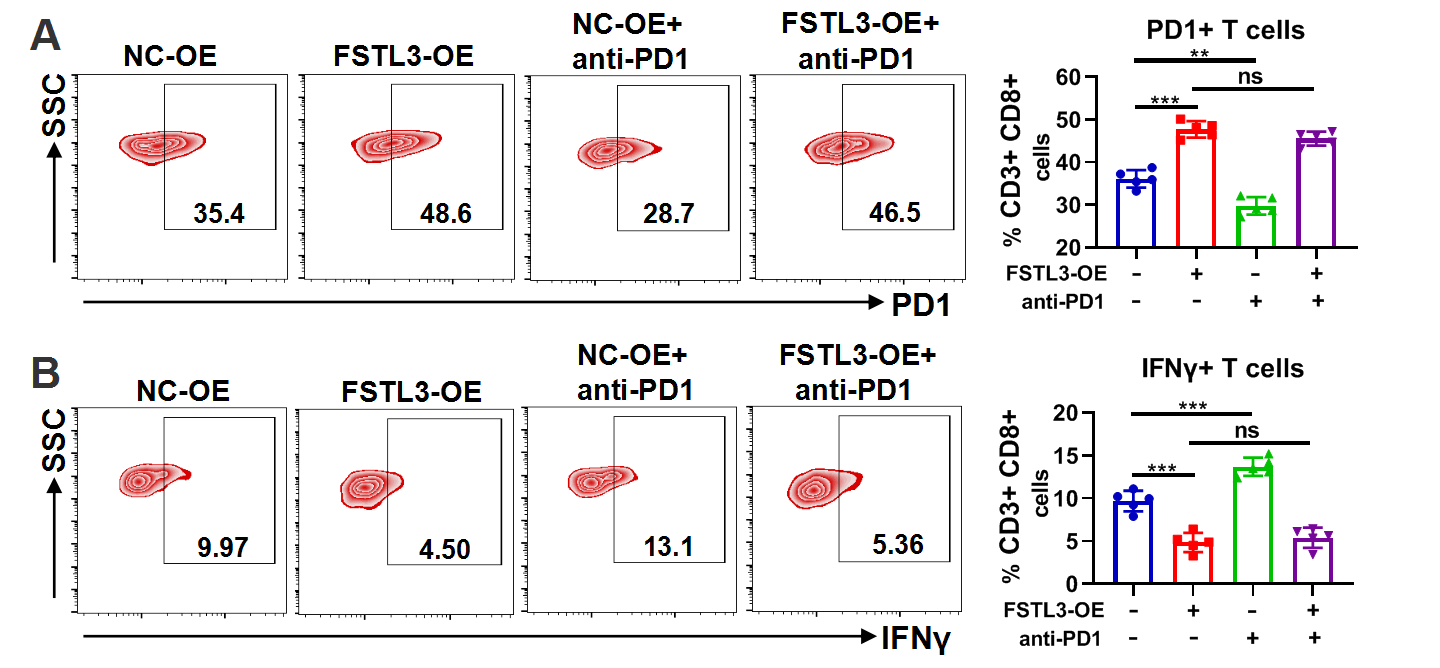


**Supplementary Figure S7. Flow cytometry analysis of CD8^+^ T cells in mouse tumors established with NC-OE or FSTL3-OE MC38 cells and treated with anti-PD1 antibody.** **(A-B)** Flow cytometry analysis of PD1 (A) and IFNγ (B) in CD3^+^ CD8^+^ T cells in tumor tissues of C57BL/6J mice in each group. Data are shown as mean ± SD. ***P* < 0.01, ****P* < 0.001, ns, non-significant.

**Supplementary Figure S8**

**
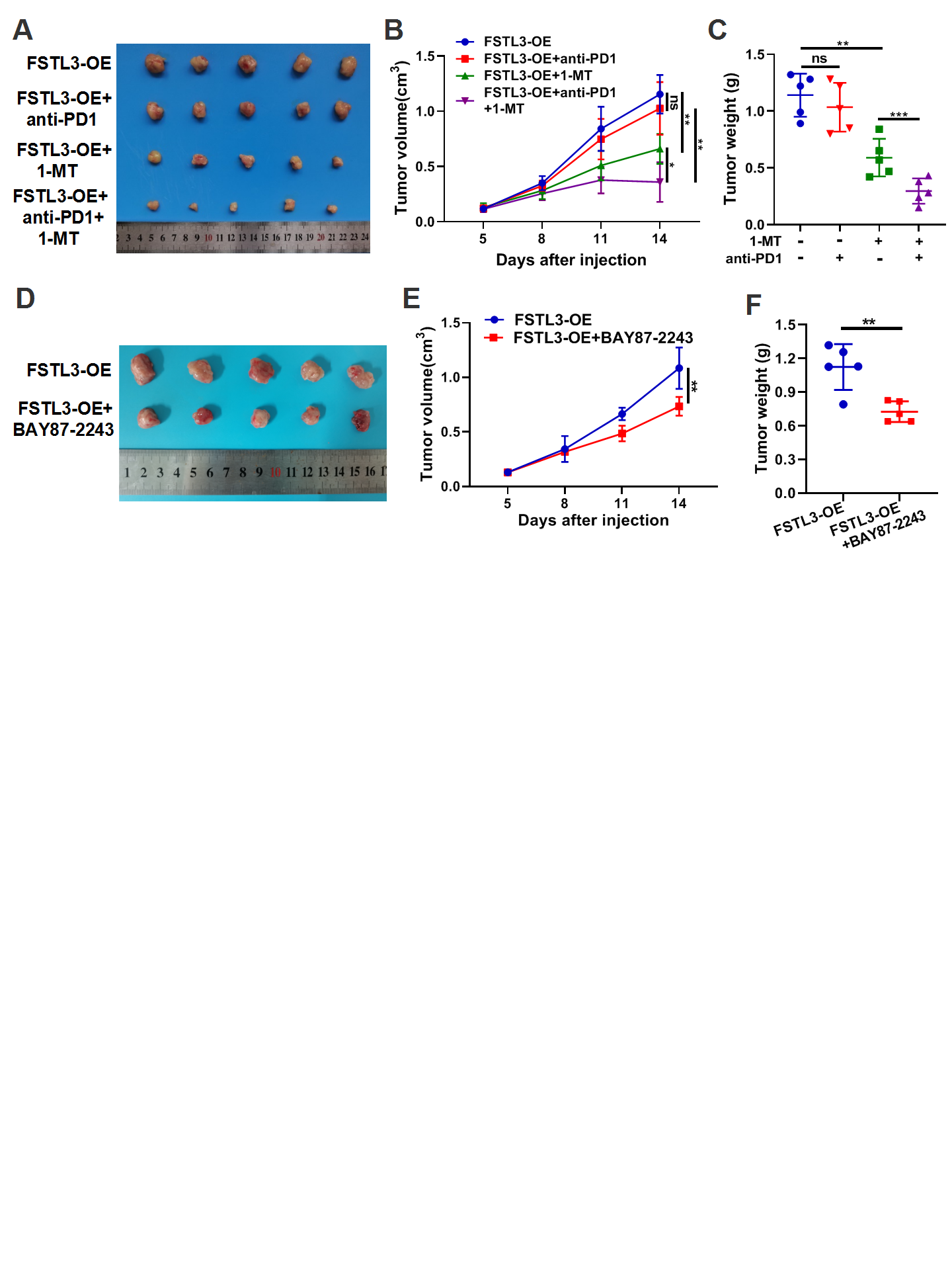
**

**Supplementary Figure S8. IDO1 inhibitor and HIF1α inhibitor inhibit FSTL3 high-expressing tumor growth.** **(A-C)** The mice were injected subcutaneously with FSTL3-OE MC38 cells to establish tumor models, followed by combined treatment with anti-PD1 antibody and 1-MT. Tumor images (A), growth curves (B) and weight (C) were obtained at day 14 after dissection. **(D-F)** The mice were injected subcutaneously with FSTL3-OE MC38 cells to establish tumor models, followed by treatment with BAY87-2243. Tumor images (D), growth curves (E) and weight (F) were obtained at day 14 after dissection. Data are shown as mean ± SD. **P* < 0.05, ***P* < 0.01, ****P* < 0.001, ns, non-significant.
