# Supplementary material for: FSTL3 promotes tumor immune evasion and attenuates response to anti-PD1 therapy by stabilizing c-Myc in colorectal cancer
Source: Cell Death Dis. 2024 Feb 1;15(2):107. doi: 10.1038/s41419-024-06469-0 (PMC10834545; doi:10.1038/s41419-024-06469-0)
Supplement: Supplementary file 2 — Supplementary tables [file 41419_2024_6469_MOESM2_ESM.docx]

**Supplementary Tables**

**Supplementary Table S1.** Correlation between FSTL3 expression and clinicopathological characteristics of CRC patients.

| Variable | Cases  (n = 95) | FSTL3 expression | | *P* values |
| --- | --- | --- | --- | --- |
|  |  | Low (n = 50) | High (n = 45) |  |
| Age (years) |  |  |  |  |
| <60 | 40 | 21 | 19 | 0.983 |
| ≥60 | 55 | 29 | 26 |  |
| Gender |  |  |  |  |
| Female | 35 | 17 | 18 | 0.545 |
| Male | 60 | 33 | 27 |  |
| Tumor size |  |  |  |  |
| <5 cm | 40 | 28 | 12 | 0.004 |
| ≥5 cm | 55 | 22 | 33 |  |
| T stage |  |  |  |  |
| T1-2 | 20 | 17 | 3 | 0.004 |
| T3 | 61 | 28 | 33 |  |
| T4 | 14 | 5 | 9 |  |
| N stage |  |  |  |  |
| N0 | 51 | 32 | 19 | 0.034 |
| N1 | 44 | 18 | 26 |  |
| M stage |  |  |  |  |
| M0 | 74 | 44 | 30 | 0.012 |
| M1-2 | 21 | 6 | 15 |  |
| TNM stage |  |  |  |  |
| Stage I | 11 | 7 | 4 | 0.004 |
| Stage II | 39 | 28 | 11 |  |
| Stage III | 36 | 13 | 23 |  |
| Stage IV | 9 | 2 | 7 |  |

**Supplementary Table S2.** Antibodies used for for chromatin immunoprecipitation (ChIP), immunoprecipitation (IP), immunohistochemical (IHC), immunofluorescence (IF) and western blotting (WB).

| Name | Brand | Cat. No. | Dilution |
| --- | --- | --- | --- |
| Rabbit Anti-FSTL3 antibody | Bioss | bs-16188R | 1:200(IF)  1:200(IHC) |
| FSTL3 Rabbit pAb | ABclonal | A10279 | 1:1000(WB) |
| Rabbit Anti-Cytokeratin 19 antibody | Bioss | bsm-52059R | 1:100(IF) |
| Beta Actin Polyclonal antibody | Proteintech | 20536-1-AP | 1:2500(WB) |
| HIF-1α (D1S7W) XP® Rabbit mAb | Cell Signaling Technology | 36169 | 1:100(ChIP) |
| c-MYC Polyclonal antibody | Proteintech | 10828-1-AP | 1:3000(WB)  1:150(IF)  1:80(ChIP)  2µg/400µg protein (IP) |
| DYKDDDDK tag Monoclonal antibody | Proteintech | 66008-3-Ig | 1:1000(WB)  2µg/400µg protein (IP) |
| PD-L1/CD274 Monoclonal antibody | Proteintech | 66248-1-Ig | 1:5000(WB) |
| Mouse IgG | Proteintech | B900620 | 2µg/400µg protein (IP) |
| HIF1A Antibody | Affinity | AF1009 | 1:1000(WB) |
| ubiquitin Polyclonal antibody | Proteintech | 10201-2-AP | 1:800(WB) |
| Anti-indoleamine 2,3-deoxygenase antibody | Abcam | ab211017 | 1:1000(WB) |
| HA-Tag (C29F4) Rabbit mAb | Cell Signaling Technology | 3724 | 1:1000(WB)  1:50(IP) |
| Rabbit Anti-CD8 antibody | Bioss | bs-0648R | 1:200(IHC) |
| Rabbit Anti-FOXP3 antibody | Bioss | bs-10211R | 1:200(IHC) |
| F4/80 Polyclonal antibody | Proteintech | 28463-1-AP | 1:4000(IHC) |
| Rabbit Anti-CD163 antibody | Bioss | bs-2527R | 1:200(IHC) |
| Mouse Anti-CD206 antibody | Bioss | bsm-60762M | 1:200(IHC) |
| HRP-conjugated Affinipure Goat Anti-Rabbit IgG(H+L) | Proteintech | SA00001-2 | 1:5000(WB) |
| HRP-conjugated Affinipure Goat Anti-Mouse IgG(H+L) | Proteintech | SA00001-1 | 1:5000(WB) |
| CoraLite488-conjugated Goat Anti-Rabbit IgG(H+L) | Proteintech | SA00013-2 | 1:200(IF) |
| CoraLite594-conjugated Goat Anti-Rabbit IgG(H+L) | Proteintech | SA00013-4 | 1:200(IF) |

**Supplementary Table S3.** Target sequences for siRNAs and shRNAs.

| Species | Name | Target sequence (5’- 3’) |
| --- | --- | --- |
| Homo | shFSTL3 | GAGCTTTGCGGCAACAACAAC |
| Homo | si-c-Myc | CCAUAAUGUAAACUGCCUCAA |
| Mus | shFSTL3 | CCCAGACTTGCGCGTCATGTA |

**Supplementary Table S4.** Sequences of forward and reverse primers used for qRT-PCR and ChIP.

| Species | Gene | Forward Primer | Reverse Primer |
| --- | --- | --- | --- |
| Homo | ACTB | CTACCTCATGAAGATCCTCCGA | TTCTCCTTAATGTCACGCACGATT |
| Homo | HIF1A | GAACGTCGAAAAGAAAAGTCTCG | CCTTATCAAGATGCGAACTCACA |
| Homo | FSTL3 | ACTGCGTGGTGTGTCGAG | TGGCACGAGGAGATGTAGGT |
| Homo | CD274 | TGGCATTTGCTGAACGCATTT | TGCAGCCAGGTCTAATTGTTTT |
| Homo | MYC | GGCTCCTGGCAAAAGGTCA | CTGCGTAGTTGTGCTGATGT |
| Homo | IDO1 | GCCAGCTTCGAGAAAGAGTTG | ATCCCAGAACTAGACGTGCAA |
| Mus | ACTB | GGCTGTATTCCCCTCCATCG | CCAGTTGGTAACAATGCCATGT |
| Mus | FSTL3 | CACCTACCGGGACGAATGC | CCTGAGCGCAAGACTTTTGAC |
| Homo | FSTL3  (ChIP) | GGCTCAGACCACACTTGGAC | TGAGGTAACGGGGACCCAAG |
| Homo | IDO1  (ChIP) | TGAGAGTTTAGGACTGCAGCCT | CCAGAAAGGCCTGAAGGAAAACA |

**Supplementary Table S5.** Antibodies used for flow cytometry.

| Name | Brand | Cat. No. |
| --- | --- | --- |
| PE anti-human CD274 (B7-H1, PD-L1) Antibody | BioLegend | 329705 |
| FITC anti-human FOXP3 Antibody | BioLegend | 320105 |
| PE anti-human CD25 Antibody | BioLegend | 356103 |
| CD45 Monoclonal Antibody (30-F11), PE-Cyanine7 | eBioscience | 25-0451-82 |
| IFN gamma Monoclonal Antibody (XMG1.2), APC | eBioscience | 17-7311-81 |
| APC anti-mouse CD279 (PD-1) Antibody | BioLegend | 135209 |
| CD8a Monoclonal Antibody (53-6.7), PE | eBioscience | 12-0081-82 |
| CD3 Monoclonal Antibody (17A2), PerCP-eFluor™ 710 | eBioscience | 46-0032-82 |
| FOXP3 Monoclonal Antibody (FJK-16s), APC | eBioscience | 17-5773-82 |
| PE anti-mouse CD4 Antibody | BioLegend | 100407 |
| CD25 Monoclonal Antibody (PC61.5), PerCP-Cyanine5.5 | eBioscience | 45-0251-82 |
