# Supplementary material for: FSTL3 promotes tumor immune evasion and attenuates response to anti-PD1 therapy by stabilizing c-Myc in colorectal cancer
Source: Cell Death Dis. 2024 Feb 1;15(2):107. doi: 10.1038/s41419-024-06469-0 (PMC10834545; doi:10.1038/s41419-024-06469-0)
Supplement: Supplementary file 3 — Full and uncropped western blots [file 41419_2024_6469_MOESM3_ESM.pptx]

## Slide 1
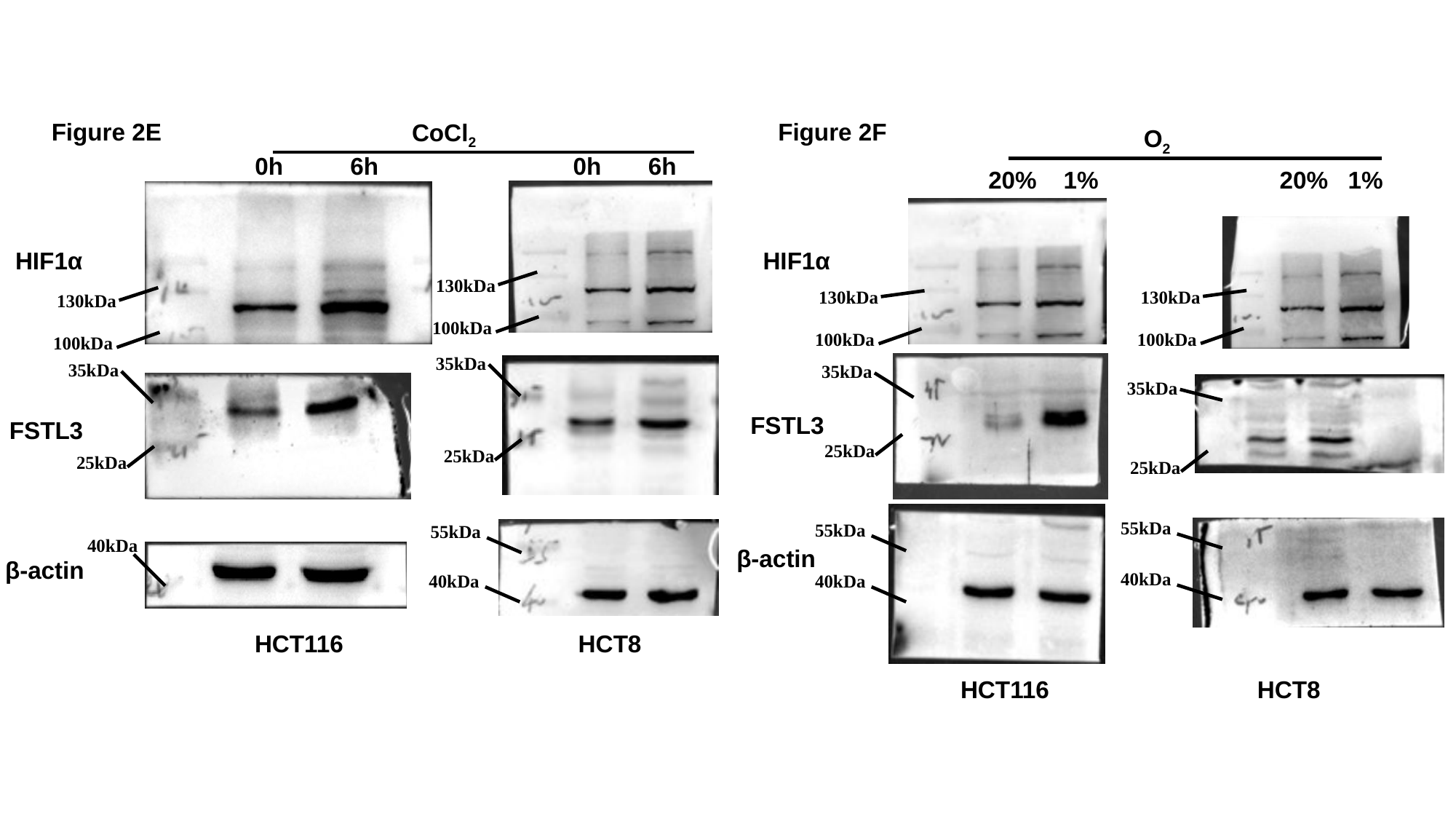

Figure 2F
Figure 2E
 CoCl2
 O2
 0h 6h 0h 6h
 20% 1% 20% 1%
HIF1α
HIF1α
130kDa
130kDa
130kDa
130kDa
100kDa
100kDa
100kDa
100kDa
35kDa
35kDa
35kDa
35kDa
FSTL3
FSTL3
25kDa
25kDa
25kDa
25kDa
55kDa
55kDa
55kDa
40kDa
β-actin
β-actin
40kDa
40kDa
40kDa
 HCT116 HCT8
 HCT116 HCT8

## Slide 2
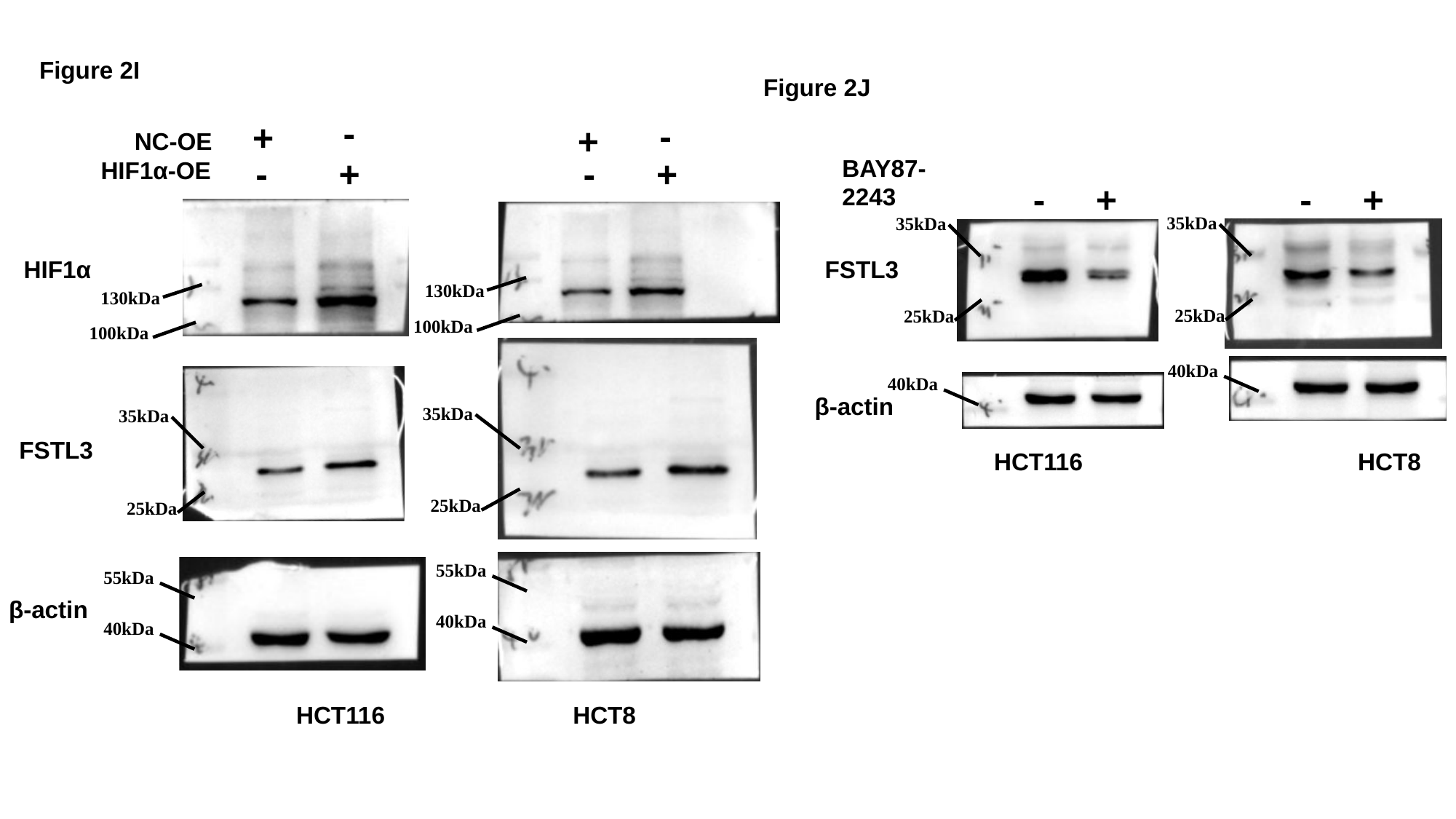

Figure 2I
Figure 2J
 -
 -
 +
 +
 NC-OE
 HIF1α-OE
 - + - +
BAY87-
2243
 - + - +
35kDa
35kDa
HIF1α
FSTL3
130kDa
130kDa
25kDa
25kDa
100kDa
100kDa
40kDa
40kDa
β-actin
35kDa
35kDa
FSTL3
 HCT116 HCT8
25kDa
25kDa
55kDa
55kDa
β-actin
40kDa
40kDa
 HCT116 HCT8

## Slide 3
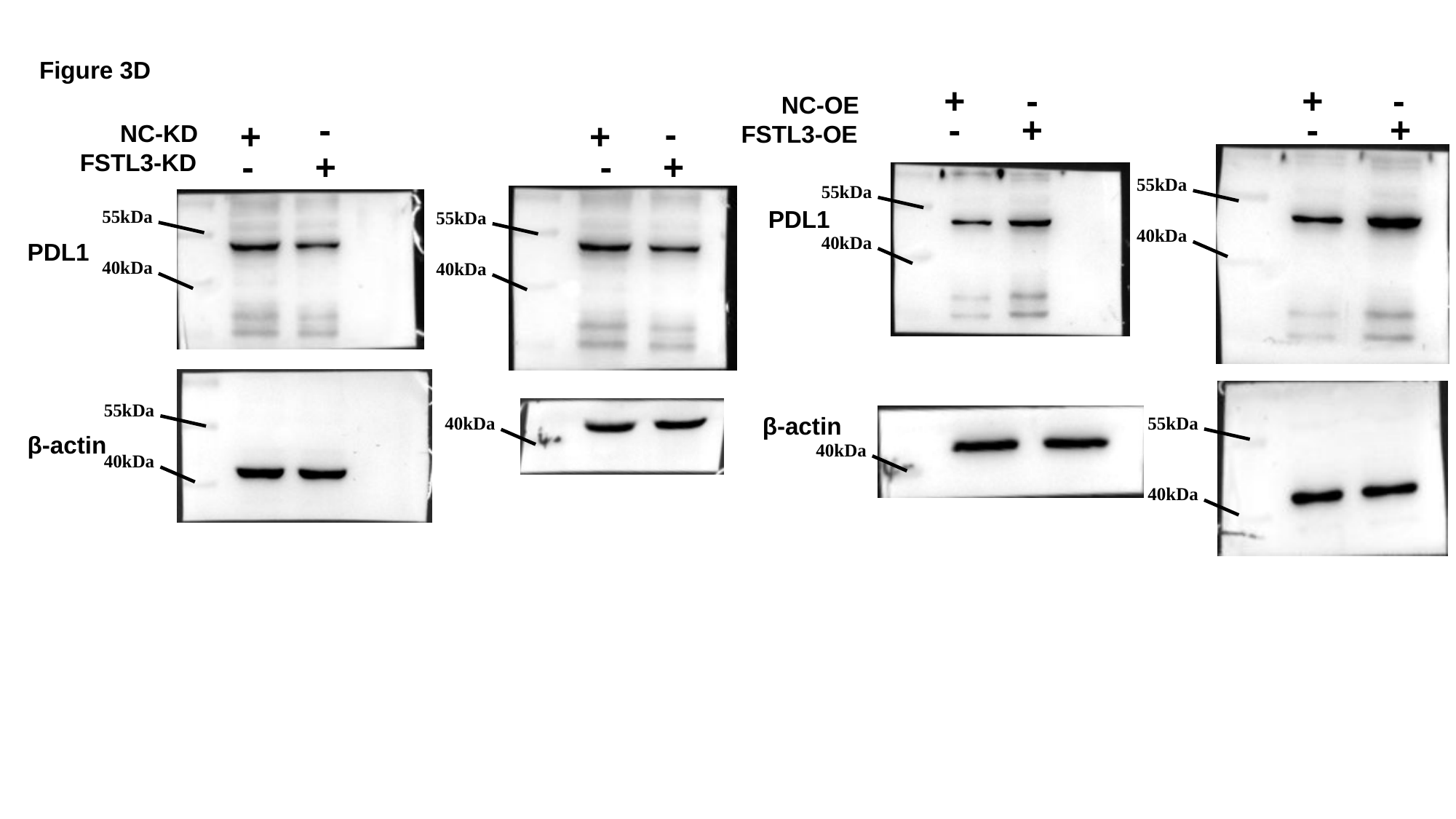

Figure 3D
 + - + -
 NC-OE
 FSTL3-OE
 -
 - + - +
 -
 +
 +
 NC-KD
 FSTL3-KD
 - + - +
55kDa
55kDa
PDL1
55kDa
55kDa
40kDa
40kDa
PDL1
40kDa
40kDa
55kDa
β-actin
55kDa
40kDa
β-actin
40kDa
40kDa
40kDa

## Slide 4
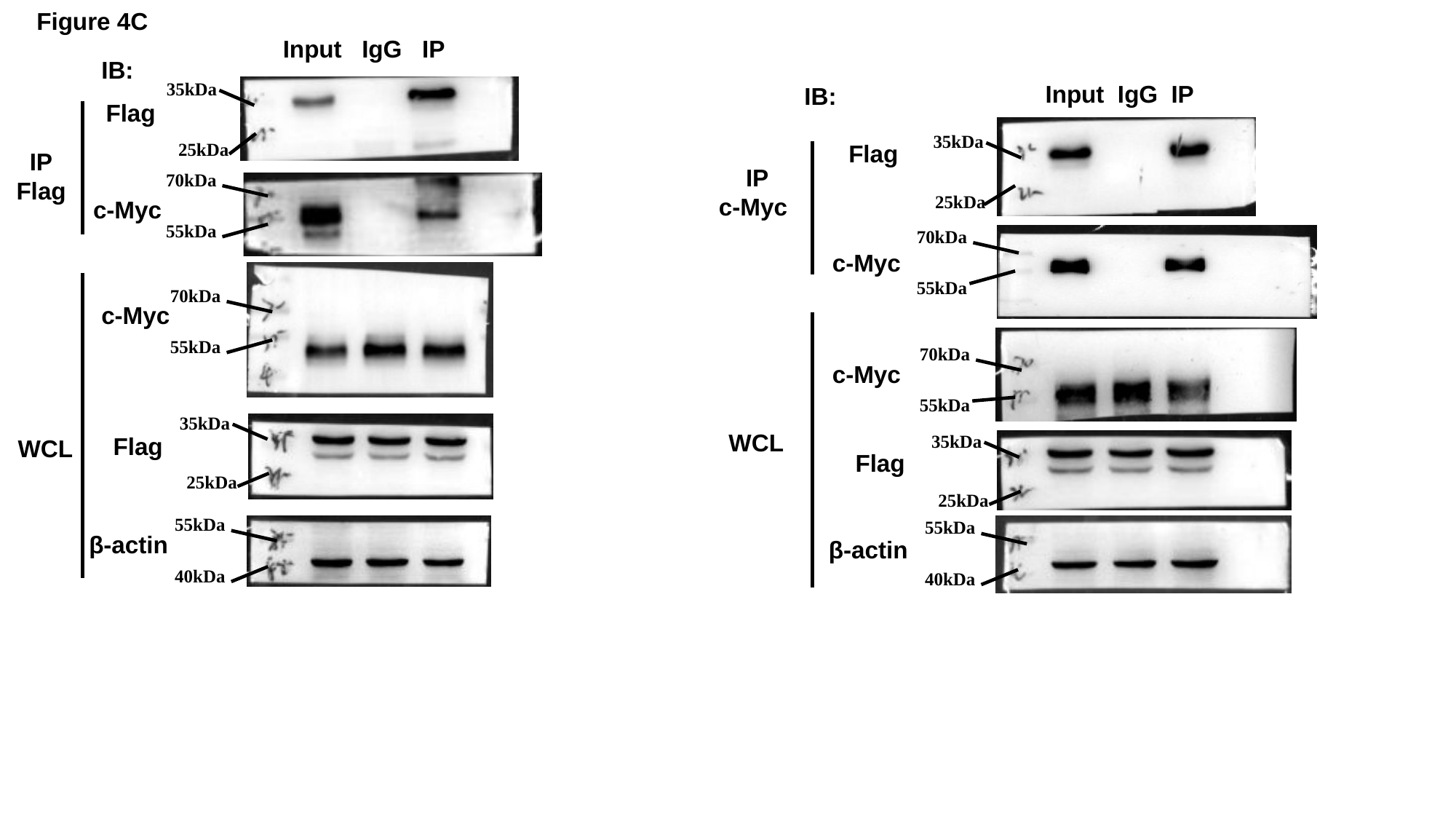

Figure 4C
Input IgG IP
IB:
35kDa
Input IgG IP
IB:
Flag
35kDa
25kDa
Flag
 IP
c-Myc
c-Myc
c-Myc
 Flag
 β-actin
 IP
Flag
70kDa
25kDa
c-Myc
55kDa
70kDa
55kDa
70kDa
c-Myc
55kDa
70kDa
55kDa
35kDa
WCL
35kDa
Flag
WCL
25kDa
25kDa
55kDa
55kDa
β-actin
40kDa
40kDa

## Slide 5
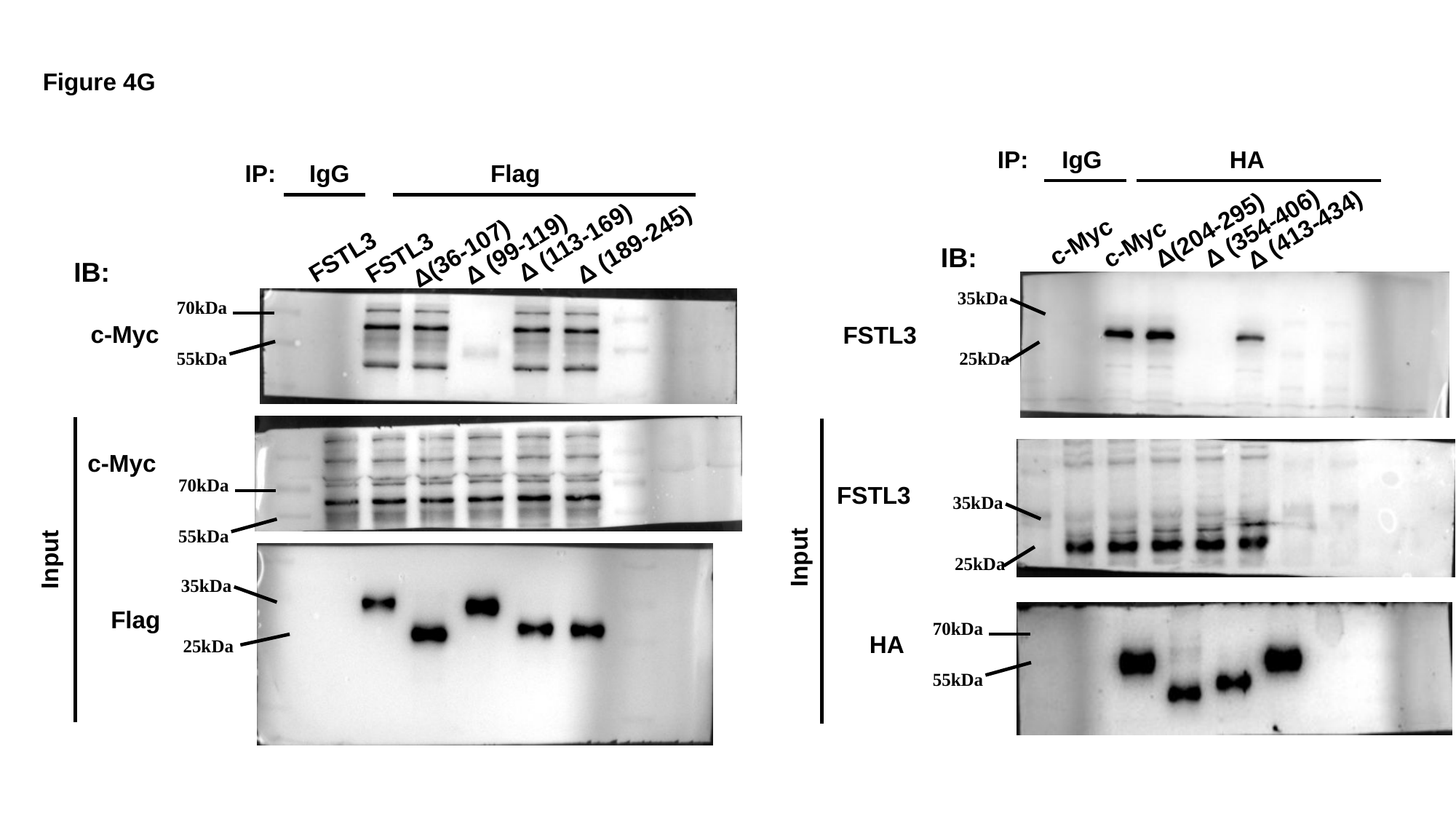

Figure 4G
 IP: IgG HA
c-Myc
Δ (354-406)
Δ (413-434)
c-Myc
Δ(204-295)
 IB:
 IP: IgG Flag
Δ (113-169)
Δ (189-245)
Δ (99-119)
Δ(36-107)
FSTL3
FSTL3
 IB:
c-Myc
c-Myc
Input
Flag
35kDa
70kDa
FSTL3
FSTL3
Input
HA
55kDa
25kDa
70kDa
35kDa
55kDa
25kDa
35kDa
70kDa
25kDa
55kDa

## Slide 6
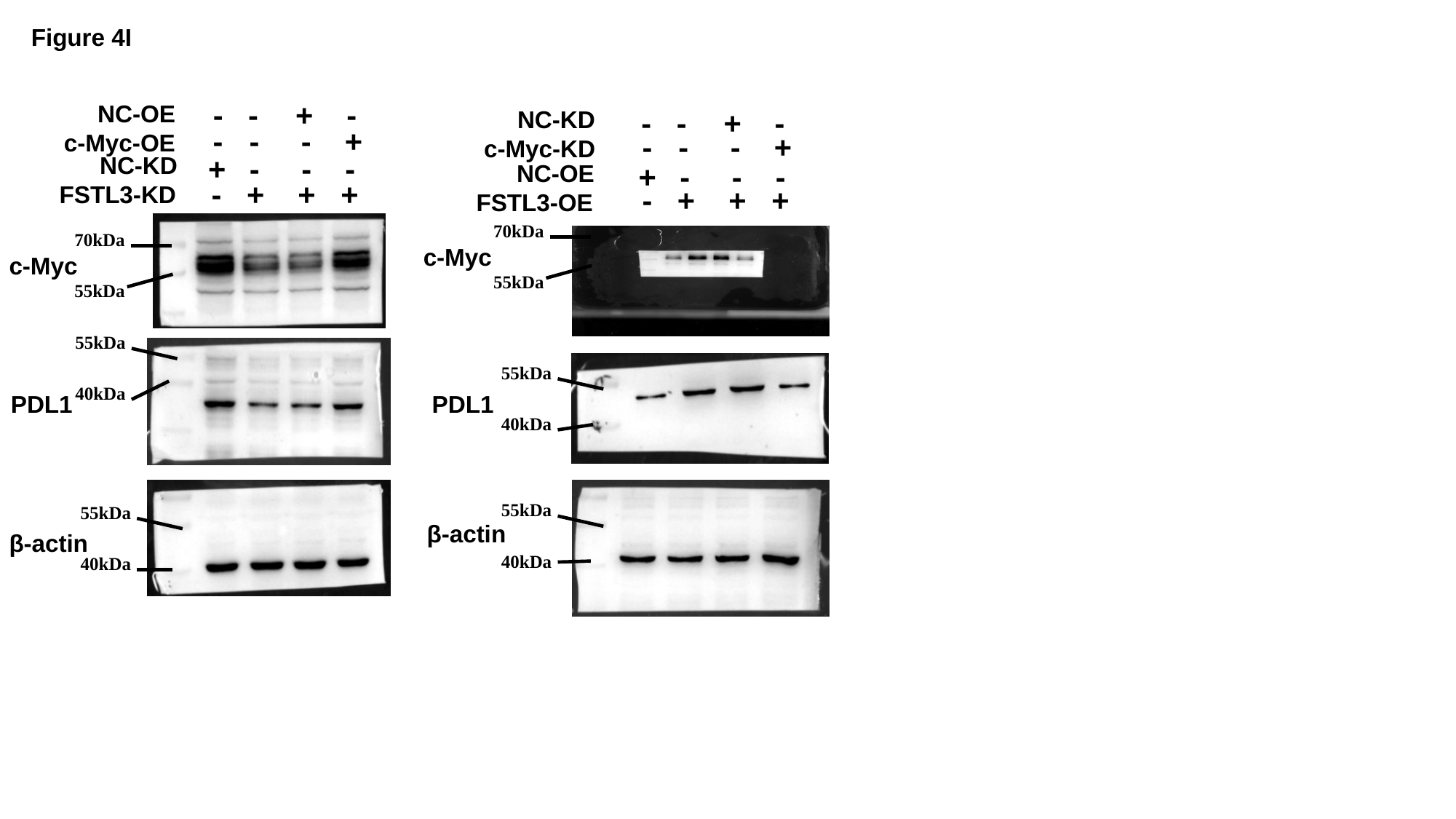

Figure 4I
 - - + -
 NC-OE
 c-Myc-OE
 NC-KD
 FSTL3-KD
c-Myc
β-actin
 - - + -
 NC-KD
 c-Myc-KD
 NC-OE
 FSTL3-OE
c-Myc
β-actin
 - - - +
 - - - +
 + - - -
 + - - -
 - + + +
 - + + +
70kDa
70kDa
55kDa
55kDa
55kDa
55kDa
40kDa
PDL1
PDL1
40kDa
55kDa
55kDa
40kDa
40kDa

## Slide 7
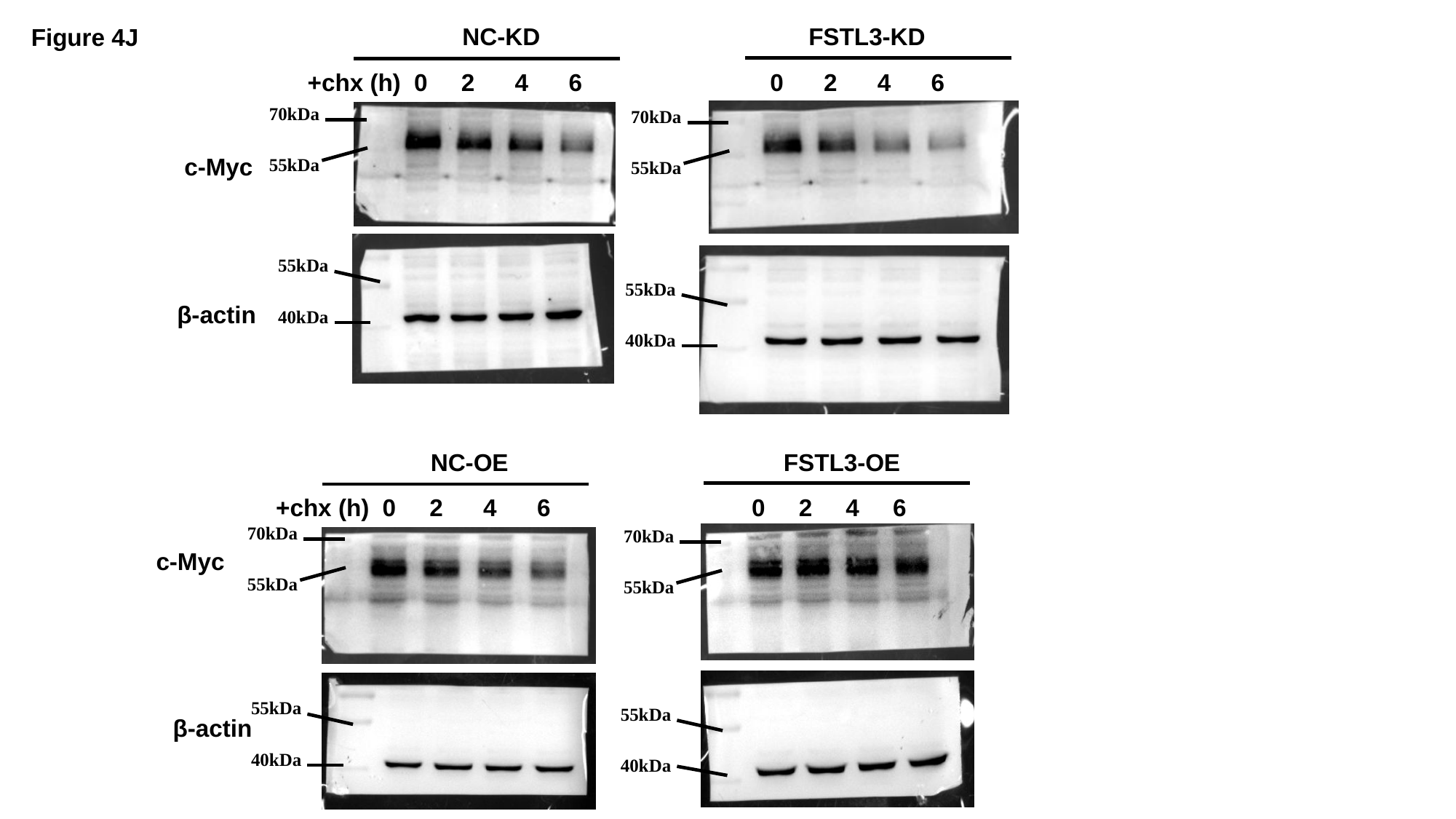

NC-KD FSTL3-KD
c-Myc
β-actin
+chx (h) 0 2 4 6 0 2 4 6
Figure 4J
70kDa
70kDa
55kDa
55kDa
55kDa
55kDa
40kDa
40kDa
 NC-OE FSTL3-OE
c-Myc
β-actin
+chx (h) 0 2 4 6 0 2 4 6
70kDa
70kDa
55kDa
55kDa
55kDa
55kDa
40kDa
40kDa

## Slide 8
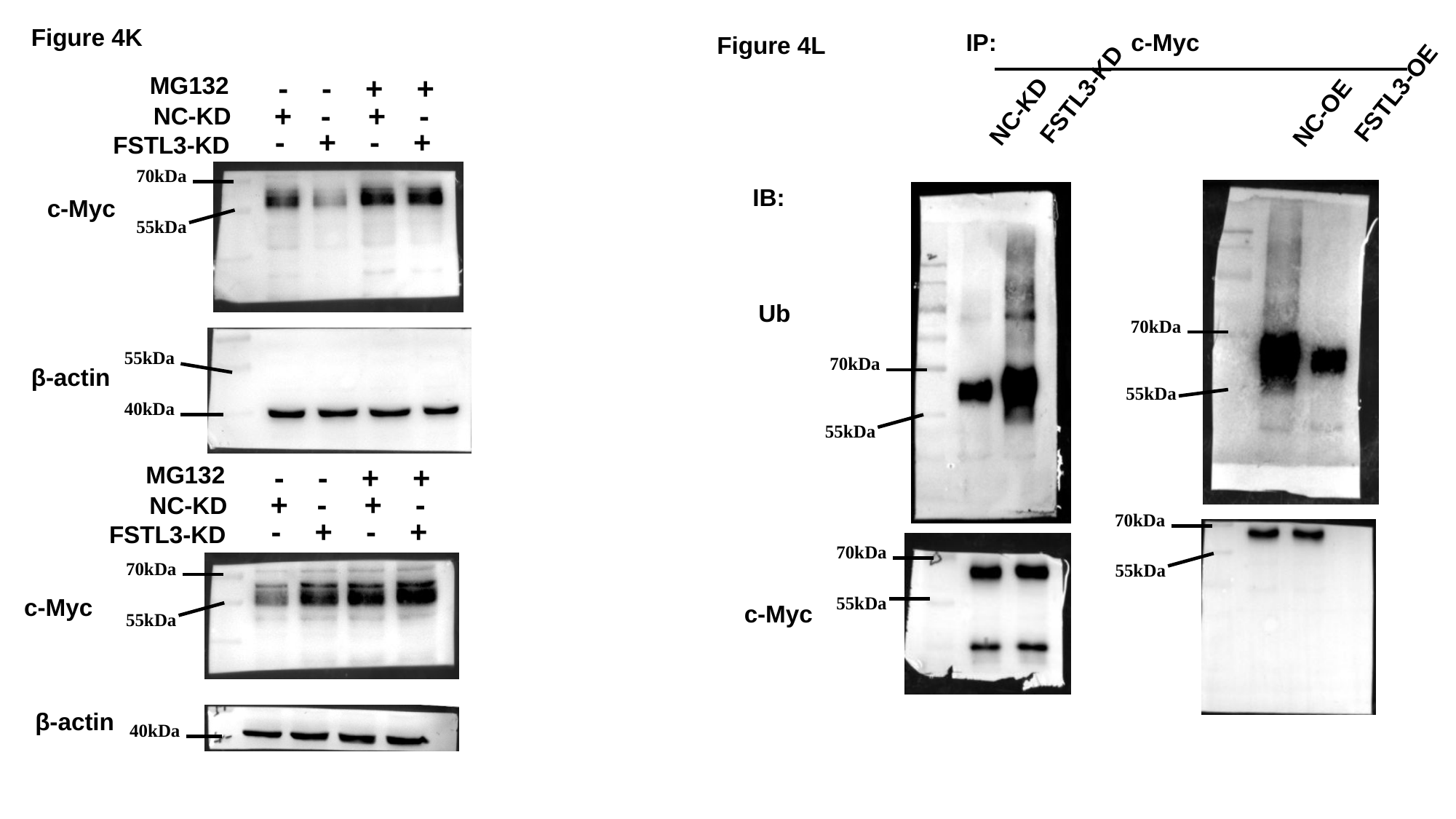

IP: c-Myc
FSTL3-OE
FSTL3-KD
NC-KD
NC-OE
IB:
Ub
c-Myc
Figure 4K
Figure 4L
 - - + +
MG132
 NC-KD
 FSTL3-KD
c-Myc
β-actin
 + - + -
 - + - +
70kDa
55kDa
70kDa
55kDa
70kDa
55kDa
40kDa
55kDa
 - - + +
MG132
 NC-KD
 FSTL3-KD
c-Myc
β-actin
 + - + -
 - + - +
70kDa
70kDa
70kDa
55kDa
55kDa
55kDa
40kDa

## Slide 9
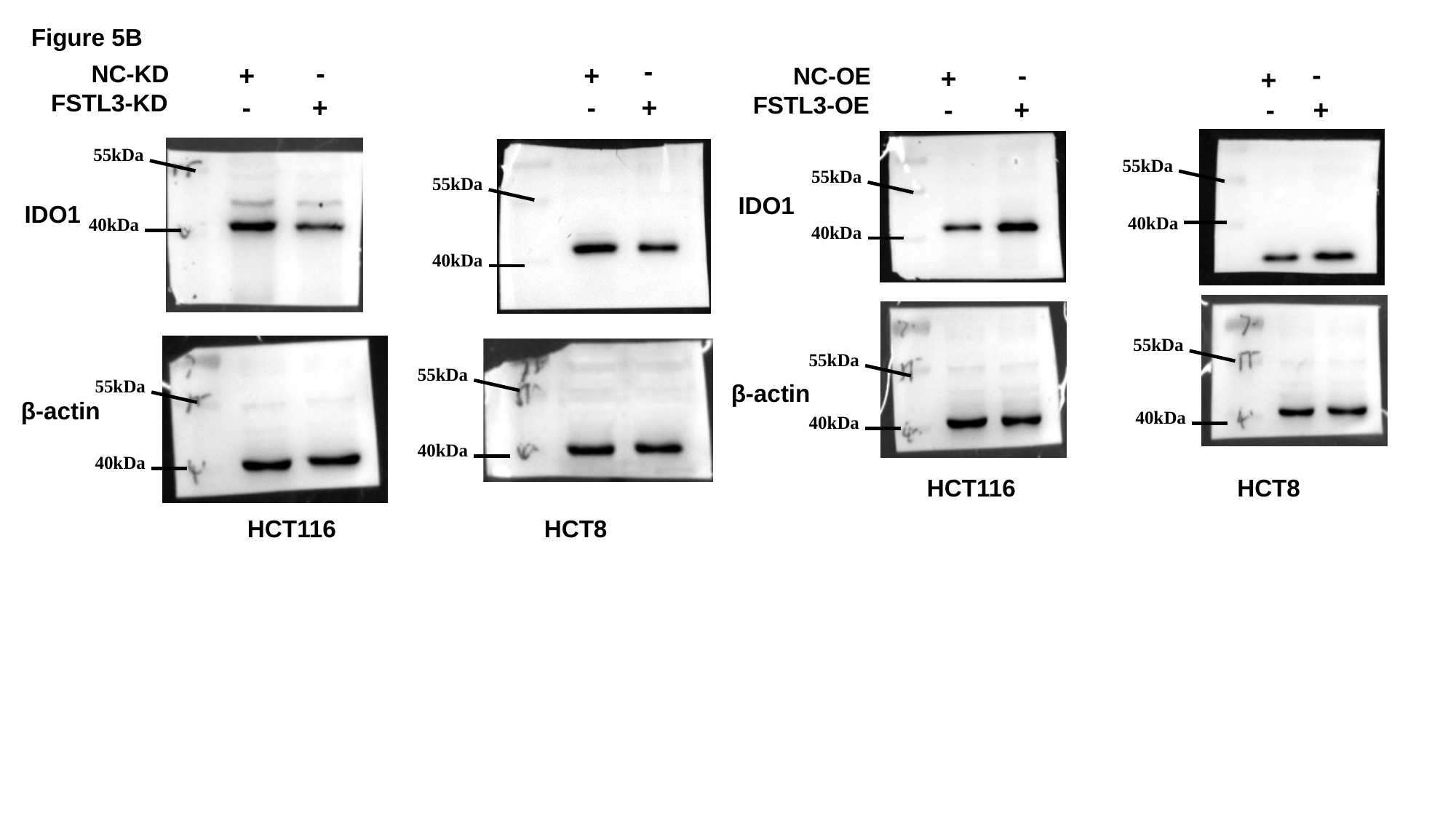

Figure 5B
 -
 -
 NC-KD
 FSTL3-KD
 +
 +
 - + - +
IDO1
β-actin
 HCT116 HCT8
 -
 -
 NC-OE
 FSTL3-OE
 +
 +
 - + - +
IDO1
β-actin
 HCT116 HCT8
55kDa
55kDa
55kDa
55kDa
40kDa
40kDa
40kDa
40kDa
55kDa
55kDa
55kDa
55kDa
40kDa
40kDa
40kDa
40kDa

## Slide 10
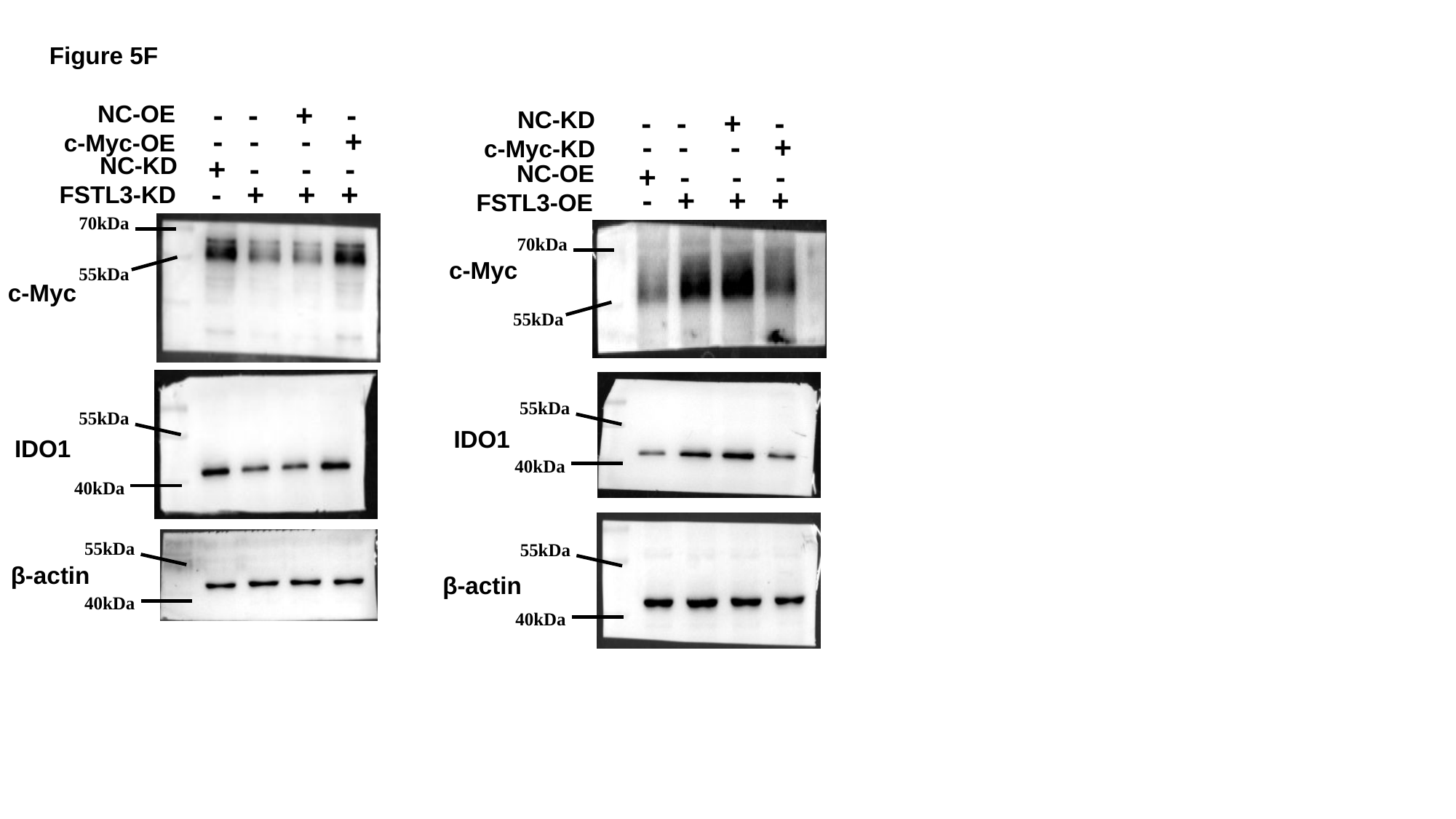

Figure 5F
 - - + -
 NC-OE
 c-Myc-OE
 NC-KD
 FSTL3-KD
c-Myc
β-actin
 - - + -
 NC-KD
 c-Myc-KD
 NC-OE
 FSTL3-OE
c-Myc
β-actin
 - - - +
 - - - +
 + - - -
 + - - -
 - + + +
 - + + +
70kDa
70kDa
55kDa
55kDa
55kDa
55kDa
IDO1
IDO1
40kDa
40kDa
55kDa
55kDa
40kDa
40kDa

## Slide 11
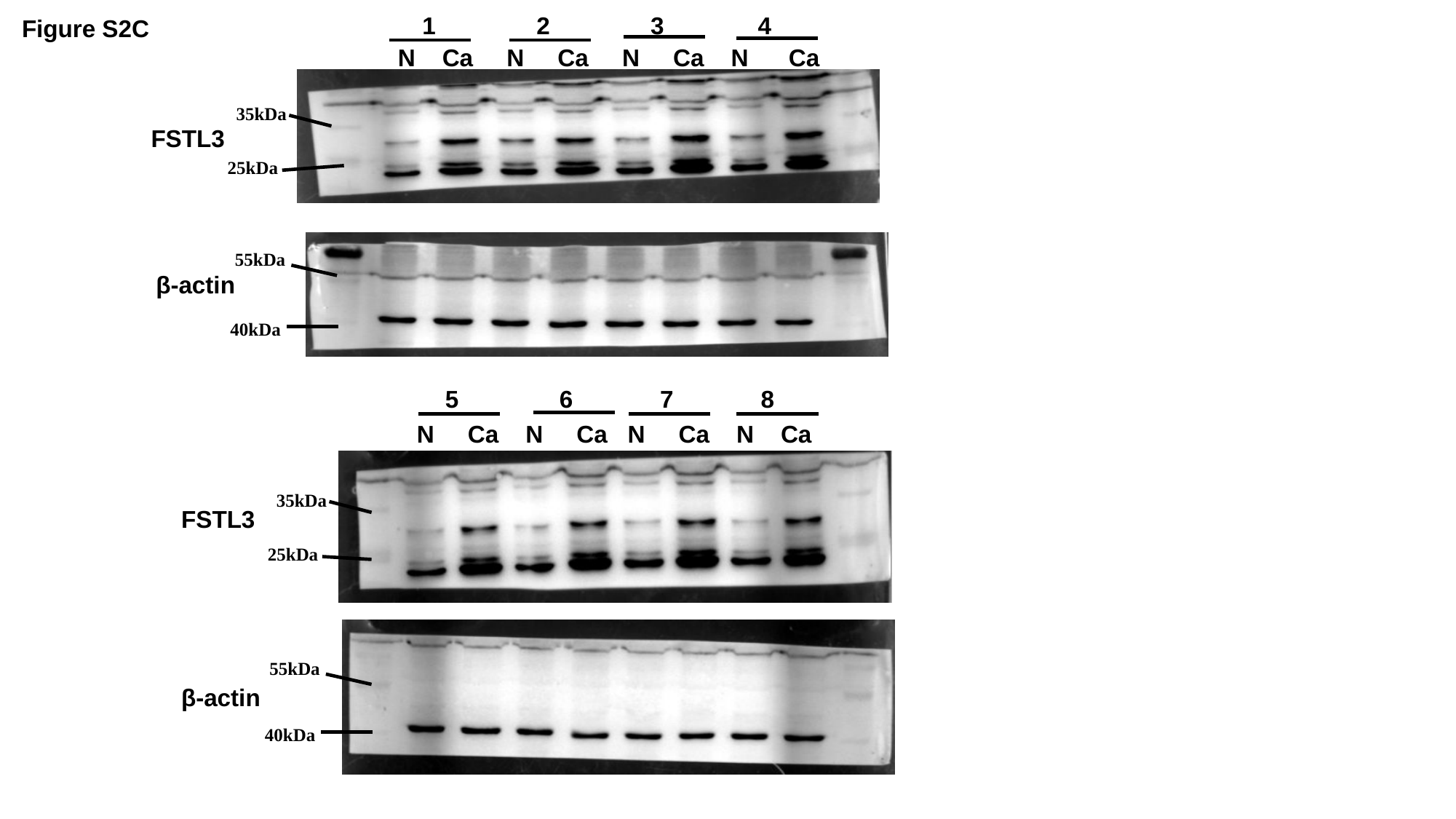

1 2 3 4
 N Ca N Ca N Ca N Ca
FSTL3
β-actin
Figure S2C
35kDa
25kDa
55kDa
40kDa
 5 6 7 8
 N Ca N Ca N Ca N Ca
FSTL3
β-actin
35kDa
25kDa
55kDa
40kDa

## Slide 12
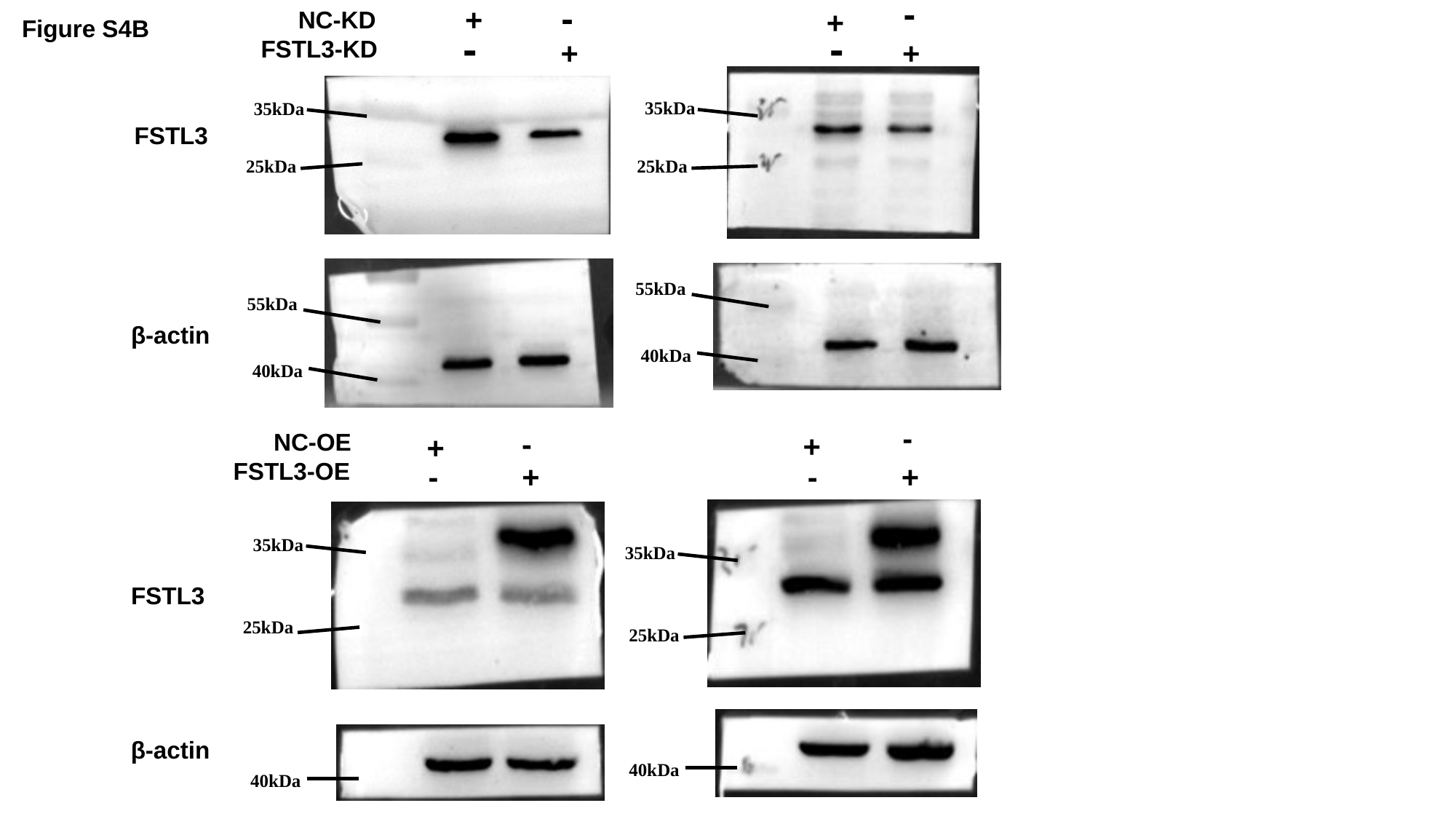

+
 -
 +
 -
 NC-KD
 FSTL3-KD
 - + - +
FSTL3
β-actin
Figure S4B
35kDa
35kDa
25kDa
25kDa
55kDa
55kDa
40kDa
40kDa
 -
 -
 NC-OE
 FSTL3-OE
 +
 +
 - + - +
FSTL3
β-actin
35kDa
35kDa
25kDa
25kDa
40kDa
40kDa

## Slide 13
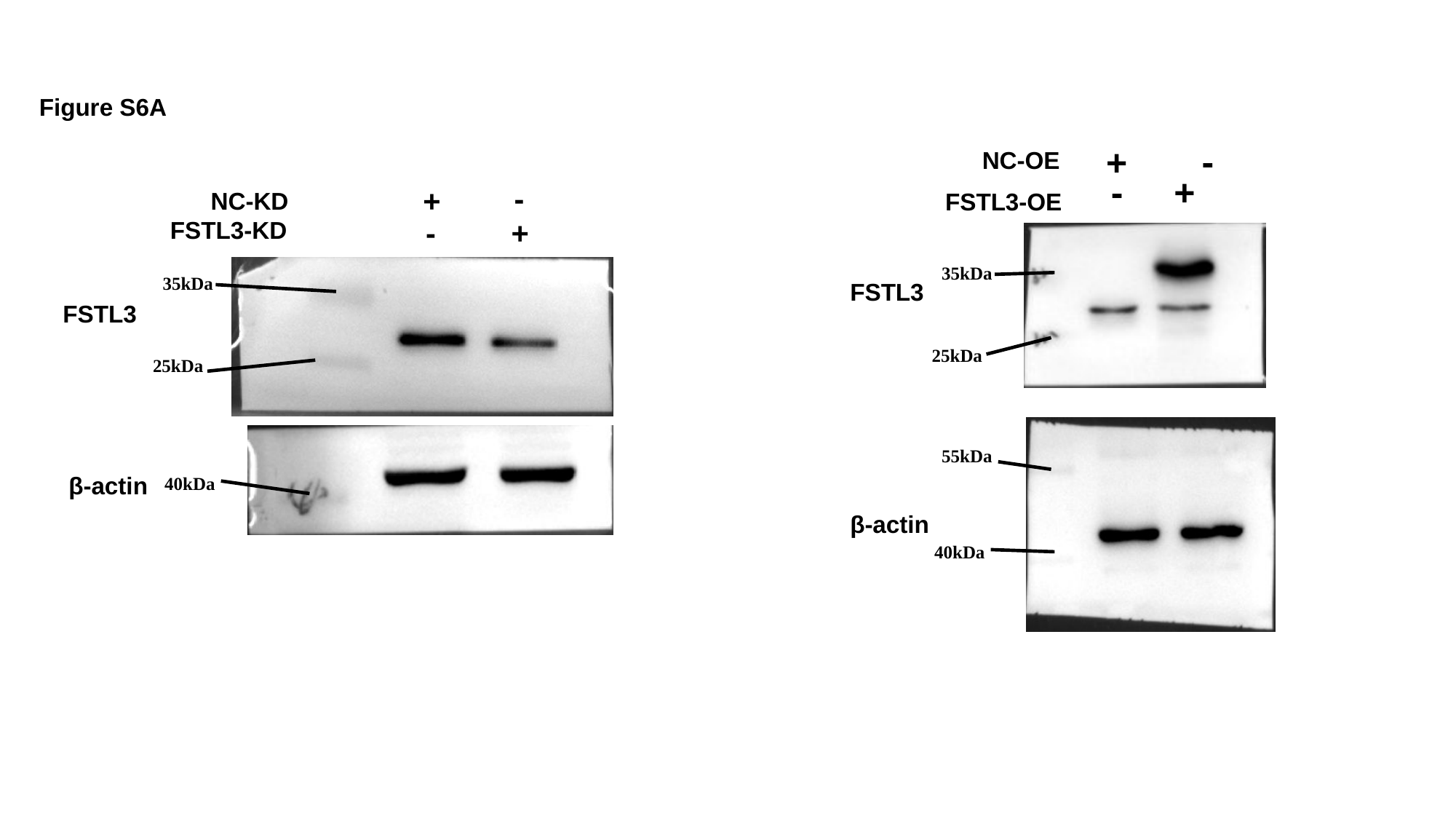

Figure S6A
 -
 +
 NC-OE
 FSTL3-OE
 - +
FSTL3
β-actin
 -
 +
 NC-KD
 FSTL3-KD
 - +
FSTL3
β-actin
35kDa
35kDa
25kDa
25kDa
55kDa
40kDa
40kDa
